# Age-Related Changes in Brain Structure in Pediatric Chronic Kidney Disease

**DOI:** 10.1001/jamanetworkopen.2024.57601

**Published:** 2025-02-03

**Authors:** Ellen van der Plas, Eric Nelson, Brian Becknell, Anne E. Dawson, Camille S. Wilson, Jeffrey D. Dawson, Joseph L. Alge, Lyndsay A. Harshman

**Affiliations:** 1 Department of Pediatrics, University of Arkansas for Medical Sciences, Little Rock; 2 Division of Hematology and Oncology, Arkansas Children’s Hospital Research Institute, Little Rock; 3 Center for Biobehavioral Health, Nationwide Children’s Hospital, Columbus, Ohio; 4 Department of Pediatrics, Ohio State University, Columbus; 5 Kidney and Urinary Tract Center, Abigail Wexner Research Institute, Nationwide Children’s Hospital, Columbus, Ohio; 6 Department of Pediatric Psychology and Neuropsychology, Nationwide Children’s Hospital, Columbus, Ohio; 7 Department of Biostatistics, University of Iowa, Iowa City; 8 Stead Family Department of Pediatrics, University of Iowa Health Care, Iowa City

## Abstract

**Importance:**

Pediatric patients with chronic kidney disease (CKD) exhibit reduced cerebellum volume, which is associated with neurocognitive deficits and a lower estimated glomerular filtration rate (eGFR), even before dialysis or transplantation. These differences have not been examined within the context of age-related brain changes during childhood to early adulthood.

**Objective:**

To evaluate differences in age-related neurodevelopmental changes in patients with CKD compared with control participants and to investigate associations between regional neuroanatomy, functional outcomes, and disease-related variables.

**Design, Setting, and Participants:**

Case-control study of individuals aged 6 through 21 years with and without CKD at an academic medical center in Iowa City, Iowa, from September 2016 to August 2024.

**Exposures:**

Neurocognitive testing; 3-T magnetic resonance imaging.

**Main Outcomes and Measures:**

Participants completed standardized neurocognitive assessments and quantitative neuroanatomical scans. Brain regions of interest (ROIs) were analyzed for volumetric differences using automated pipelines. Multivariable linear models assessed neurocognitive and neuroanatomical differences between groups, including an age × group interaction for ROI analyses.

**Results:**

The sample included 124 individuals (mean [SD] age, 12.8 [4.5] years; 74 [59.7%] male), including 87 control participants (44 [50.6%] male) and 37 participants with CKD (30 [81.1%] male). The mean (SD) eGFR was 71.3 [25.5] mL/min/1.73 m^2^ for the CKD group. Participants with CKD scored lower than control participants on most neurocognitive measures included in the analyses. The CKD group showed differential age-related changes in cerebellar gray matter (β = −0.10; 95% CI, −0.18 to −0.01; Cohen *f* = 0.22) and white matter (β = −0.09; 95% CI, −0.19 to −0.00; Cohen *f* = 0.19). The age × group interaction approached but did not reach significance for amygdala volume (β = 0.09; 95% CI, −0.01 to 0.19; Cohen *f* = 0.18; *P* = .06). Volumetric variation in these regions was associated with proxy ratings of executive function in patients with CKD. A significant, positive association between cerebellar gray matter and eGFR was observed in the CKD group (β = 0.04; 95% CI, 0.00 to 0.02; *P* = .01).

**Conclusions and Relevance:**

In this case-control study, age-related neurodevelopmental differences were observed in pediatric patients with CKD compared with healthy peers. Reductions in cerebellar volume were associated with cognitive deficits and lower kidney function. These findings underscore the importance of monitoring neurodevelopmental trajectories in children with CKD, as early interventions may be necessary to mitigate cognitive impairments associated with CKD.

## Key Points

**Question:**

What do age-related changes in the brain look like in children, adolescents, and young adults with and without chronic kidney disease (CKD)?

**Findings:**

In this case-control study including 124 individuals, patients with CKD showed different age-related changes in cerebellum gray and white matter volume compared with unaffected controls. Abnormal cerebellum gray matter was associated with executive dysfunction and lower kidney function.

**Meaning:**

These findings suggest CKD is associated with abnormal cerebellar growth, which may explain neurocognitive difficulties observed in this population.

## Teaser

This case-control study evaluates differences in age-related neurodevelopmental changes in children, adolescents, and young adults with chronic kidney disease compared with control participants.

## Introduction

Pediatric chronic kidney disease (CKD) is characterized by progressive decline in kidney function and lifelong multisystem disease sequelae. Approximately 50% of pediatric CKD diagnoses are due to congenital anomalies of the kidney and urinary tract (CAKUT).^1^ Half of these patients may require kidney transplantation (KT) during their lifetime.^2^ In pediatric CKD, decline in kidney function and associated comorbidities—including acidosis, hypertension, and anemia—occur simultaneously with dynamic neurodevelopment and neurocognitive maturation.^3,4,5^ Patients with CKD are at risk for neurocognitive deficits, which develop before need for dialysis or transplantation and may not improve following pediatric KT.^6,7^ Deficits are commonly reported in executive functioning (EF),^8^ cognitive processes necessary for attention regulation and planning. EF deficits worsen with lower kidney function,^8,9,10^ longer CKD duration,^9,11,12^ metabolic acidosis,^13,14^ and hypertension,^15^ suggesting an effect of CKD on neuronal systems mediating EF.

Adolescence and young adulthood reflect peak periods of neurocognitive maturation, including development of EF networks.^16^ There are brain white matter (WM) volume increases, reduction in gray matter (GM) density, and enhanced organization of brain functional networks.^3,17,18,19^ Although EF is often associated with prefrontal brain regions, the cerebellum is implicated in complex cognitive control processes associated with EF.^20,21,22,23,24^ Similar to neocortical regions, the cerebellum undergoes a protracted development characterized by an increase of GM during adolescence, followed by a decrease of GM volume.^4,5^

We previously found^25^ that children with CKD due to CAKUT have reduced cerebellar GM volume compared with unaffected controls. Those with the lowest kidney function had lower cerebellar GM volume. Cerebellar volumetric differences were present before dialysis or transplantation and were associated with poorer EF performance.^25^ Our prior work was limited to males, without assessment of age-related neurodevelopmental trends extending into early adulthood. It is critical to consider neurocognitive and neurodevelopmental differences in pediatric CKD within the context of expected neurodevelopmental changes. The primary objectives of this study were to (1) explore age-related neurodevelopmental trajectories in adolescent and young adult patients with CKD, and (2) assess age-related trends in regional brain volumes in relation to neurocognitive outcomes and disease parameters within patients with CKD.

## Methods

This case-control study was approved by the institutional review board at the University of Iowa. Written informed consent and assent was obtained from participants and legal guardians. The study followed the Strengthening the Reporting of Observational Studies in Epidemiology (STROBE) reporting guidelines.

Potentially eligible participants with CKD were identified at the University of Iowa through electronic medical records (EMR) between September 2016 and August 2024. Study inclusion and exclusion criteria were designed to recruit a homogenous CKD cohort given that previously published neuroimaging data suggest a lack of observed effect due to heterogeneity in recruited CKD samples.^26^ eFigure 1 and eTable 1 in Supplement 1 display inclusion and exclusion criteria for the CKD cohort.^25^ Potential participants with CKD were excluded before participation if any of the following were identified: extreme prematurity (<30 weeks’ gestational age), seizures and/or receipt of antiepileptic medications, central nervous system anomalies, known chromosomal anomalies, congenital cardiac disease, diagnosed intellectual disability, traumatic brain injury requiring hospitalization, and/or magnetic resonance imaging (MRI) contraindications.

Unaffected controls were recruited from the local community via advertisements with intent to align controls with participants with CKD based on age, sex, and maternal education. This group was enrolled to represent a spectrum of both normal brain development and presumed normal kidney function. Exclusion criteria were otherwise as per the CKD group, with additional exclusion of those in talented and gifted programs to mitigate the risk of confounding cognitive differences between groups.

### Study Procedures

Participants completed neurocognitive assessment and structural MRI on the day of the research visit. Anthropometric data and blood samples were obtained. Parents or caregivers completed surveys including participant and family demographics; participant birth, developmental, and medical history; participant educational needs; and family medical history. Parental socioeconomic status (SES) was reported using the Hollingshead Four Factor Index of Socioeconomic Status.^27^

### Neurocognitive Assessments

Participants completed age-appropriate, standardized neurocognitive assessments with a focus on EF (eTable 2 in Supplement 1). Tests were administered by trained examiners and supervised by a licensed psychologist (see acknowledgments).

Scores were converted to *z* scores (mean [SD], 0 [1]) using the psychometric conversion table. The Behavior Rating Inventory of Executive Function (BRIEF)^28^ yields *t* scores whereby higher scores represent poorer performance. For consistency, *z* scores derived from *t* scores were inverted so that high scores represented better performance across measures.

### Disease-Related Parameters

Kidney function was estimated from blood samples using the combined creatinine-cystatin C CKiD U25 equation.^29^ Serum bicarbonate level (mEq/L) and hemoglobin (g/dL) were obtained for case and controls. Proteinuria (mg/mg) was obtained for cases. As CKD cause was CAKUT, patient age was a proxy for disease duration. Medication and disease-related histories were obtained for all participants (Table).

**Table.** Sample Characteristics

Characteristic	Patients, No. (%)		Estimated mean difference (95% CI)	*P* value
	Controls (n = 87)	CKD (n = 37)		
Sex				
Female	43 (49.4)	7 (18.9)	0.31 (0.12 to 0.49)	.003
Male	44 (50.6)	30 (81.1)		
Age, y				
Mean (SD)	13.0 (4.72)	12.4 (4.05)	−0.65 (−2.42 to 1.11)	.46
Median (range)	11.9 (6.17-25.4)	12.7 (6.25-21.8)		
CKID eGFR, mL/min/1.73 m^2^				
Mean (SD)	101 (17.3)	71.3 (25.5)	−29.89 (−37.75 to −22.02)	<.001
Median (range)	98.7 (66.1-158)	73.3 (21.4-131)		
Missing	3 (3.4)	0		
95% BP systolic, mm Hg				
Mean (SD)	118 (3.57)	117 (3.76)	−0.69 (−2.10 to 0.72)	.34
Median (range)	120 (109-124)	118 (108-124)		
95% BP diastolic, mm Hg				
Mean (SD)	77.6 (2.86)	77.2 (3.14)	−0.43 (−1.57 to 0.72)	.46
Median (range)	78.0 (70.0-83.0)	78.0 (69.0-80.0)		
Serum bicarbonate, mEq/L				
Mean (SD)	26.1 (4.61)	24.7 (2.17)	−1.36 (−2.93 to 0.22)	.09
Median (range)	25.5 (21.0-64.0)	25.0 (19.0-31.0)		
Missing	1 (1.1)	0		
Hemoglobin, g/dL				
Mean (SD)	14.4 (1.05)	14.0 (1.30)	−0.42 (−0.97 to 0.14)	.14
Median (range)	14.4 (12.6-16.7)	14.2 (11.1-16.7)		
Missing	52 (59.8)	0		
Urine protein to creatinine ratio, mg/mg				
Mean (SD)	NA	0.4 (0.7)	NA	NA
Median (range)	NA	0.1 (0.04-3.16)		
Missing	NA	11 (30)		
Socioeconomic status				
Professional/high managerial positions	61 (70.1)	18 (48.6)	0.24 (0.03to to 0.45)	.02
Semi/skilled workers	23 (26.4)	19 (51.4)		
Missing	3 (3.4)	0		
Maternal education, y				
Mean (SD)	16.0 (2.22)	15.5 (2.60)	−0.50 (−1.41 to 0.42)	.29
Median (range)	16.0 (12.0-21.0)	16.0 (11.0-21.0)		
Missing	3 (3.4)	0		
Prematurity (<37 wk gestation)				
Premature	8 (9.2)	10 (27.0)	−0.18 (−0.35 to −0.00)	.02
Full term	79 (90.8)	27 (73.0)		
CKD stage^a^				
1, eGFR >90 mL/min/1.73 m^2^	NA	9 (24.3)	NA	<.001
2, eGFR 60-89 mL/min/1.73 m^2^	NA	16 (43.2)		
3, eGFR 30-59 mL/min/1.73 m^2^	NA	11 (29.7)		
4, eGFR 15-30 mL/min/1.73 m^2^	NA	1 (2.7)		
Missing	NA	0		
Individualized education program				
No	87 (100)	35 (94.6)	−0.05 (−0.15 to 0.04)	.16
Yes	0	2 (5.4)		
Growth hormone supplementation				
No	87 (100)	35 (94.6)	−0.05 (−0.15 to 0.04)	.16
Yes	0	2 (5.4)		
Vitamin D supplementation				
No	87 (100)	24 (64.9)	−0.35 (−0.52 to −0.18)	<.001
Yes	0	13 (35.1)		
Iron supplements				
No	87 (100)	25 (67.6)	−0.32 (−0.49 to −0.15)	<.001
Yes	0	12 (32.4)		
Blood pressure medication				
No	87 (100)	26 (70.3)	−0.30 (−0.46 to −0.13)	<.001
Yes	0	5 (13.5)		
Psychotropic medication				
No	86 (98.9)	32 (86.5)	−0.12 (−0.26 to 0.01)	.01
Yes	1 (1.1)	5 (13.5)		
Anxiety/depression medication				
No	83 (95.4)	30 (81.1)	−0.14 (−0.30 to 0.01)	.03
Yes	4 (4.6)	7 (18.9)		
Alkalizing agent				
No	87 (100)	34 (91.9)	−0.08 (−0.19 to 0.03)	.04
Yes	0	3 (8.1)		

Abbreviations: BP, blood pressure; CKD, chronic kidney disease; CKID, chronic kidney disease in children; eGFR, estimated glomerular filtration rate; NA, not applicable.

SI conversion factors: To convert creatinine to micromoles per liter, multiply by 88.4; hemoglobin to grams per liter, multiply by 10.0; serum bicarbonate to millimoles per liter, multiply by 1.0.

^a^ The diagnosis of CKD requires a reduction in eGFR persisting for 3 months or more.

### Neuroimaging

Participants completed a nonsedated, noncontrast-enhanced MRI of the brain. A total of 117 of 124 scans were acquired on a GE Discovery 750-W 3-T scanner. Seven of 124 participants completed scans on a Siemens TrioTim 3-T scanner. Both were equipped with a 32-channel head coil. Anatomical T1-weighted images were acquired as follows for GE (Siemens parameters in parentheses): coronal BRAVO (MPRAGE), repetition time (TR) = 8.392 (2300) ms, time to echo (TE) = 3.184 (2.82) ms, inversion time (TI) = 450 (900) ms, flip angle = 12 (10)°, field-of-view (FOV) = 282 × 282 × 264 mm, matrix = 256 × 256 × 240. Parameters for T2-weighted were coronal, TR = 3000 (4800) ms, TE = 85.925 (430) ms, FOV = 256 × 256 × 224 mm, matrix = 256 × 256 × 160. A prospective motion correction sequence was used to correct motion artifacts.^30^ All images were processed and visually inspected by a neuroimaging specialist. Five observations (2 CKD and 3 controls) were removed from the analysis because of poor scan quality.

MRI images were processed using the BRAINSAutoWorkup pipeline to optimize tissue classification through an iterative framework, producing robust parcellation of brain regions results in a multiscanner setting.^31^ BRAINSAutoWorkup labels brain regions with a multiatlas, similarity-weighted, majority-vote procedure (joint label fusion),^32^ using a set of expert-segmented templates adapted from the Desikan-Killiany atlas^33^ and SUIT atlas^34^ for cerebellum parcellation. Brain regions included cortical and subcortical regions, separated by hemispheres and tissue type where appropriate.

To reduce the number of statistical comparisons, neuroanatomical regions of interest (ROIs) from the left and right side of the brain were summed to a combined total. ROIs vary with intracranial volume (ICV). The power proportion method was employed to adjust for nonlinear associations between ICV and ROI (see eTable 3 in Supplement 1).^35^ For visualization purposes, ROI volumes were scaled by subtracting the sample mean for the ROI from individual volumes and dividing the difference by the sample standard deviation for that ROI.

### Statistical Analysis

Group demographics and neurocognitive outcomes were evaluated with linear models on age-adjusted *z* scores using group and SES as variables. Semiskilled and skilled workers (40 participants) were collapsed into a single category as only 2 SES responses reported semiskilled workers. The group × age interaction was omitted in models on neurocognitive outcomes as these measures are age-normalized.

Age-related group differences in regional volume were evaluated using linear models with ICV-power-adjusted volumes as the dependent variables. We included an age × group interaction to evaluate if the effect of age on the ROI was dependent on group. Models included a main effect for sex, which is known to influence neurodevelopmental trajectories.^36,37^ We explored the association of SES with brain outcomes (eTable 4 in Supplement 1). Since its association was limited, SES was excluded from the models. Main effects of age and group were retained in the models. Given the interest in potential differential age-related changes between groups, we did not provide an interpretation of the main effects. To minimize the number of models, we limited analyses on associations between regional neuroanatomical volume, kidney function, and neurocognitive outcomes to regions with a significant age × group interaction. Models included regional brain volume as the outcome variable with sex, CKiD U25 eGFR, and age as variables. Since eGFR varies with age,^38^ we explored whether the interaction between age and eGFR was associated with regional volume. The interaction term was dropped if unassociated with the neuroanatomical outcome. Age was omitted in models evaluating associations between regional neuroanatomy and neurocognitive outcomes as neurocognitive scores are age-adjusted. We prioritized minimizing type II errors over type I errors, using an uncorrected threshold of *P* < .05. Analyses were performed using R version 4.2.1 (R Project for Statistical Computing). Statistical tests were 2-sided.

## Results

### Sample

The sample included 124 individuals aged 6 through 21 years (mean [SD] age, 12.8 [4.5] years; 74 [59.7%] male), including 87 control participants (44 [50.6%] male) and 37 participants with CKD (30 [81.1%] male). Mean (SD) eGFR was 71.3 (25.5) mL/min/1.73 m^2^ for the CKD group and 101 (17.3) mL/min/1.73 m^2^ among controls (β = −29.87; 95% CI, −37.75 to −22.02; *P* < .001). The Table shows demographics and kidney-specific variable.

### Cognitive Outcomes

**Figure 1.**
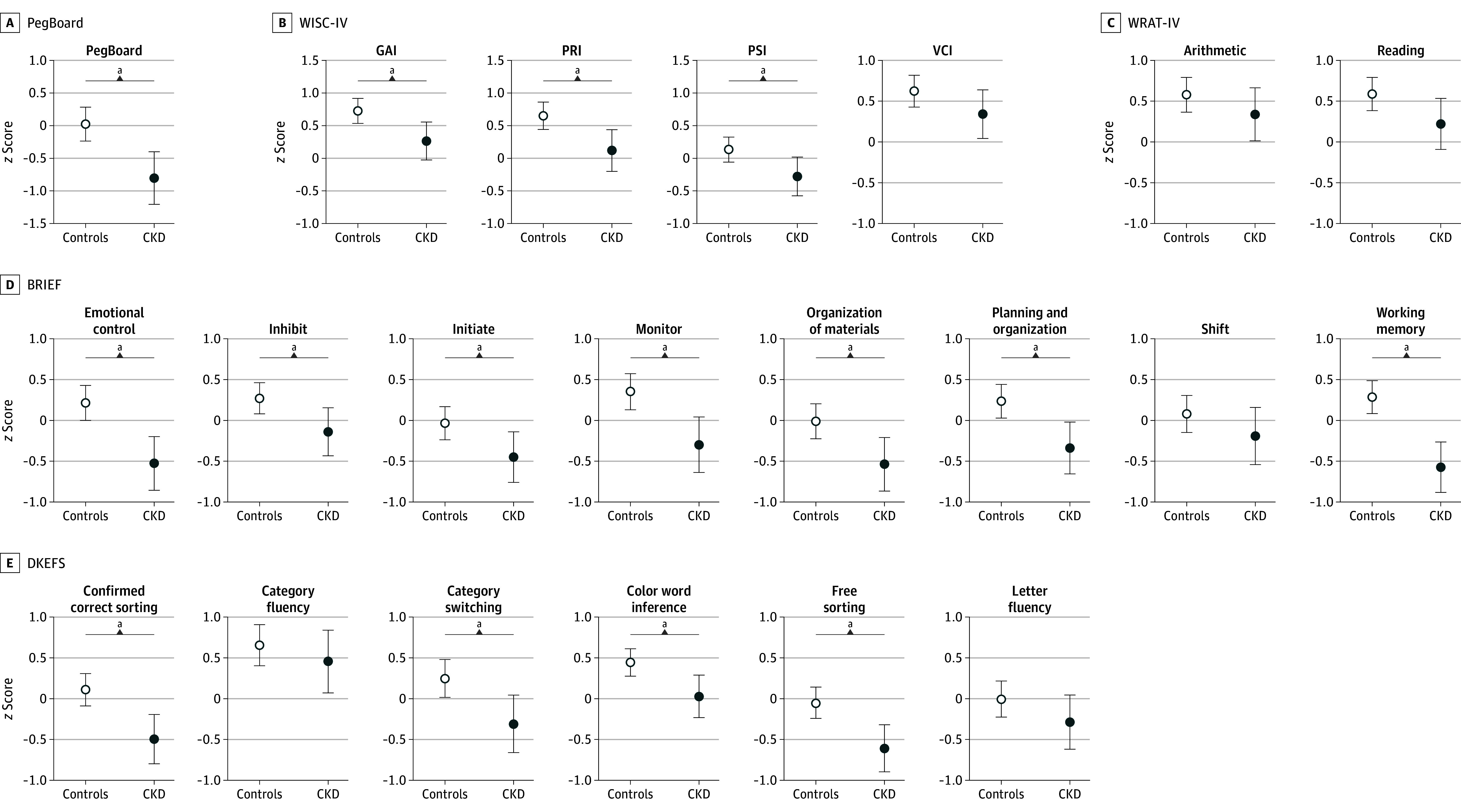
Scores on Performance-Based Measures and Parental Surveys in Patients With Chronic Kidney Disease (CKD) and Controls Each panel shows socioeconomic status–adjusted means scores and 95% confidence limits for measures listed at the top for controls (white dots) and patients with CKD (blue dots). A, PegBoard total time for the dominant hand. B, Scores for WISC-IV GAI, PRI, PSI, and VCI. C, WRAT-4 Arithmetic and Reading; D, BRIEF Emotional Control; inhibit; initiate; monitor; organization of materials; planning and organization; shift; and working memory; E, DKEFS Confirmed Sorting Correct; category fluency; category switching; color-word interference; free sorting; and letter fluency. BRIEF indicates Behavior Rating Inventory of Executive Function; DKEFS, Delis–Kaplan Executive Function System; GAI, General Ability Index; PRI, Perceptual Reasoning Index; PSI, Processing Speed Index; VCI, Verbal Comprehension Index; WISC, Wechsler Intelligence Scale for Children; WRAT, Wide Range Achievement Test. ^a^Denotes differences that were significant at *P* < .05.

The CKD group scored significantly lower than control participants on most neurocognitive measures (Figure 1). See eTable 5 in Supplement 1 for group estimates.

### Age-Related Change in Brain Outcomes

**Figure 2.**
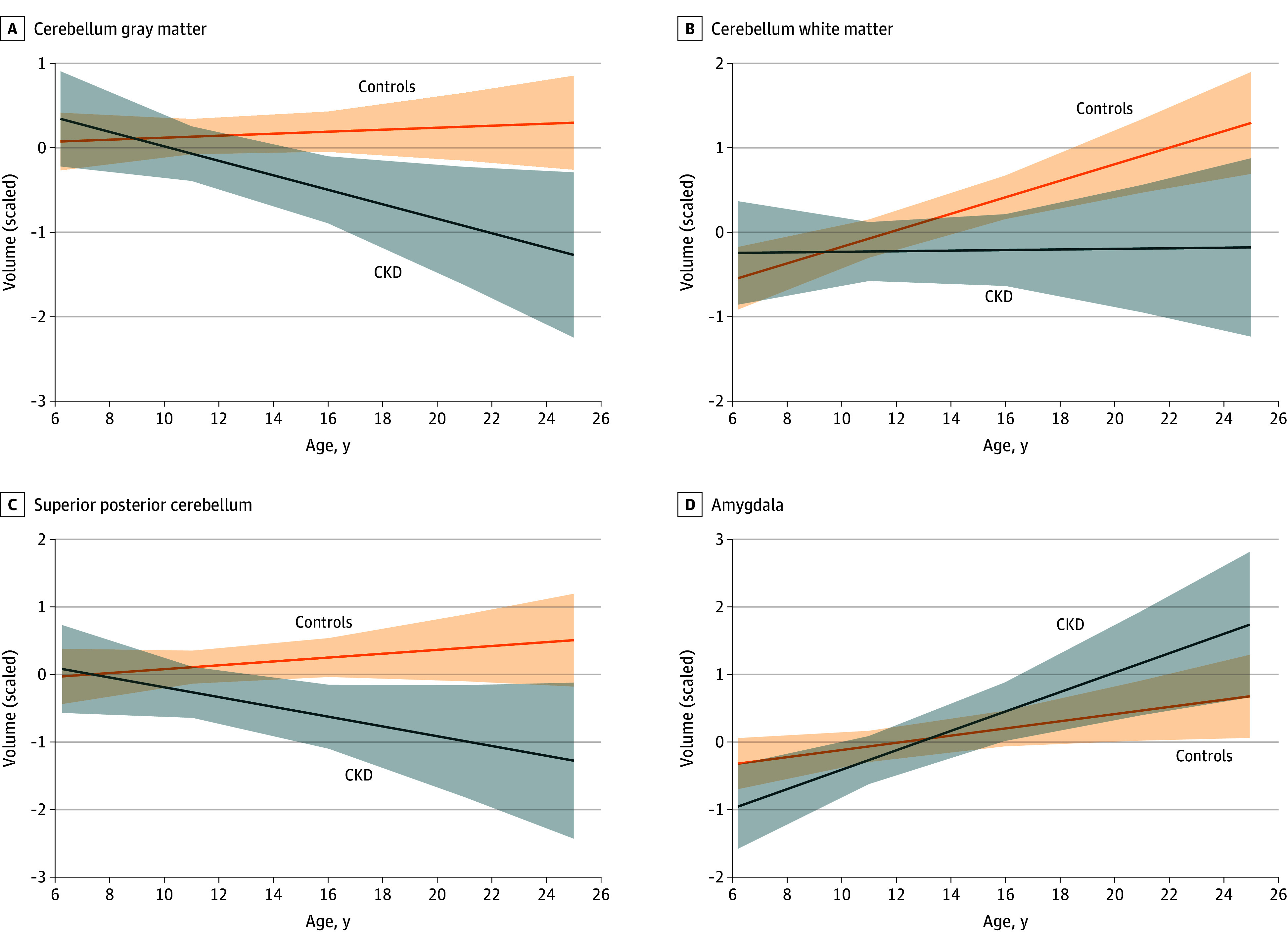
Age-Related Changes in Cerebellum Gray and White Matter, Cerebellum Superior Posterior Lobe Volume, and Amygdala Volume in Controls and Patients With Chronic Kidney Disease (CKD) Shaded areas indicate the CI of the regression.

eTable 6 in Supplement 1 lists summary statistics for the age × group interaction for ROIs and main effects of age, group, and sex. eFigure 2 in Supplement 1 illustrates the age × group interactions across ROIs. A significant age × group interaction effect was observed for cerebellum GM (β = −0.10; 95% CI, −0.18 to −0.01; F_(1,105)_ = 5.05; *P* = .03) (Figure 2A) and cerebellum WM (β = −0.09; 95% CI, −0.19 to −0.00; F_(1, 106)_ = 4.02; *P* = .048) (Figure 2B). Minimal age-related volumetric change in cerebellum GM was observed in controls, whereas the CKD group exhibited a decline in GM volume across age. Controls demonstrated age-related increases in cerebellum WM relative to limited age-related WM change in the CKD group. Post hoc analyses examining age-related trends across 5 major cerebellar regions showed a significant age × group interaction for the superior posterior lobe (F_(1, 99)_ = 3.81; *P* = .05) (Figure 2C). Summary statistics and visualization of trends for other cerebellum lobes are shown in eTable 7 and eFigure 3 in Supplement 1. The group × age interaction did not reach our threshold of significance for amygdala volume (β = 0.09; 95% CI, −0.01 to 0.19; F_(1, 106)_ = 3.54; *P* = .06). The CKD group showed an accelerated age-related increase in amygdala volume (Figure 2D).

### Structure Function Associations in CKD

**Figure 3.**
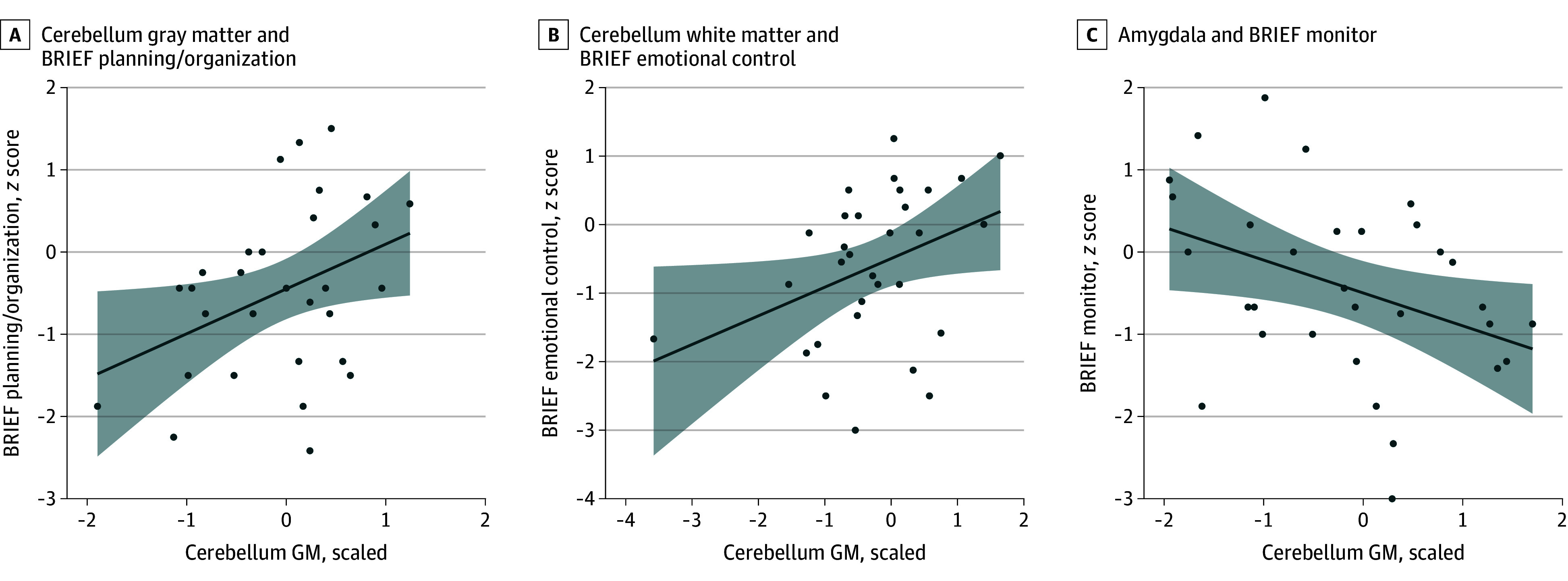
Significant Associations Between Regions of Interest and Outcomes A, Cerebellum gray matter and Behavior Rating Inventory of Executive Function (BRIEF) planning/organization; B, cerebellum white matter and BRIEF emotional control; and C, amygdala volume and BRIEF monitoring. Each point represents a participant's data, and the shading represents the CI of the regression.

Analyses were limited to cerebellum GM and WM volumes, amygdala volume, and functional (neurocognitive) outcomes with significant differences between groups (eTable 8 in Supplement 1). Associations were limited to BRIEF outcome measures, where cerebellum GM was associated with planning/organization (β = 0.55; 95% CI, 0.04 to 1.10; *P* = 04) (Figure 3A), cerebellum WM was associated with emotional control (β = 0.42; 95% CI, 0.02 to 0.82; *P* = 04) (Figure 3B), and amygdala volume was associated with monitor (β = −0.40; 95% CI, −0.76 to −0.04; *P* < .03)( Figure 3C). The cerebellum WM and emotional control association was due to an outlier, as the regression coefficient for cerebellum WM did not reach significance when this observation was removed (β = 0.48; 95% CI, −0.04 to 1.00; *P* = .07). No associations between neurocognitive outcomes and the superior-posterior lobe of the cerebellum reached significance at *P* < .05 (eTable 9 in Supplement 1).

### Disease-Related Associations With Neuroanatomical Outcomes in Patients With CKD

**Figure 4.**
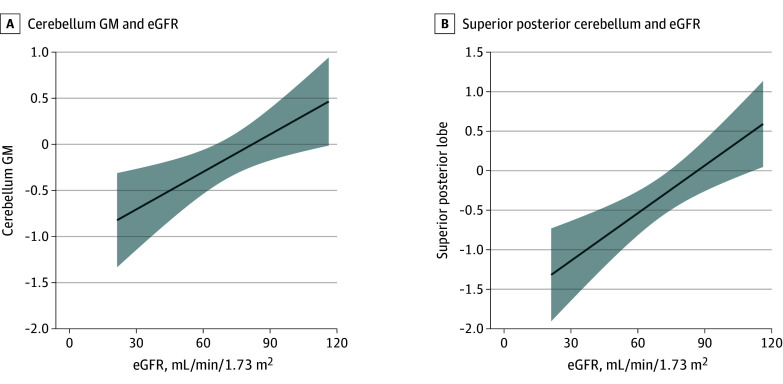
Associations Between Estimated Glomerular Filtration Rate (eGFR) and Cerebellum Volume in Patients With Chronic Kidney Disease (CKD) A, Cerebellum gray matter volume, and B, Superior posterior lobe of the cerebellum. The figure shows model-derived estimates from linear models that included CKiD U25 eGFR (shown), age and sex. GM indicates gray matter. Shaded areas indicate the CI of the regression.

The age × eGFR interaction was not associated with cerebellum GM, WM, or amygdala volume and was removed from statistical models. Age and sex were retained as main effects in the models. eGFR was associated with cerebellum GM (eGFR β = 0.01; 95% CI, 0.00 to 0.02; *P* = .01) (Figure 4A) but not cerebellum WM (eGFR β = 0.00; 95% CI, −0.01 to 0.02), nor amygdala volume (eGFR β = 0.00; 95% CI, −0.11 to 0.02). The association between eGFR and the superior posterior lobe of the cerebellum reached significance (eGFR β = 0.02; 95% CI, 0.01 to 0.03; *P* = .001) (Figure 4B). No other CKD-specific measures were associated with neuroanatomical outcomes in patients with CKD.

## Discussion

Until recently, the neurobiology of cognitive deficits in pediatric CKD was largely uncharacterized. This work extends prior findings^25,39,40^ using a larger sample encompassing young adulthood. It is the first study we know of in CKD literature to explicitly characterize age-related neurodevelopmental changes associated with CKD. We provided evidence for abnormal growth trajectories of the cerebellum in pediatric CKD; moreover, reduction in cerebellum volume was associated with both lower kidney function and EF deficits.

The cerebellum begins cellular differentiation during prenatal development and is among the last structures to mature postnatally.^41^ Its development is vulnerable to genetic and postnatal environmental stressors.^42^ The cerebellum undergoes an anteroposterior growth gradient mirroring age-related development of the cerebral cortex^43^; for example, anterior cerebellar regions that support basic sensorimotor functions mature earlier than posterior cerebellar regions associated with higher cognitive functions.^44^ Unaffected controls followed the expected growth pattern of relatively stable GM volume and increasing WM volume.^45^ In contrast, the CKD group displayed a striking decline in cerebellar GM volume that persisted into early adulthood with concomitant slowing of expected WM volume increases. Age patterns in the superior posterior lobe of the cerebellum were characterized by a GM decrease in the CKD group as opposed to a slight increase in controls. Development of subcortical structures—such as the amygdala—occurs in an inverted U-shaped developmental pattern.^45^ Amygdala volume increased as expected for young adult controls; however, there was an accelerated increase in amygdala volumes among participants with CKD.

The role of the cerebellum in nonmotor cognitive functions is now widely recognized.^46^ A poignant example of the cerebellum in nonmotor cognition is cerebellar cognitive affective syndrome, referring to a host of cognitive deficits (eg, EF, spatial cognition, and language) following injury to the superior posterior aspect of the cerebellum.^22,47,48,49,50,51,52^ We previously reported a significant association between cerebellum GM and EF as measured with the Delis–Kaplan Executive Function System Category Fluency.^25^ We again observed a similar association in patients with CKD, with lower cerebellum volume being associated with poorer performance on planning and organizing tasks from the BRIEF. An analogous trend was observed in cerebellum WM and emotional control, with the caveat of an outlier contributing to the association. A significant difference in age-related volumetric change was noted for the superior posterior cerebellum; however, it was without functional associations in this region in the CKD group. While the principle of structure determines function is generally true, King and colleagues^53^ used functional MRI to show that functional subdivisions within the cerebellum may not respect lobular boundaries. Functional MRI studies in CKD populations are needed to better understand observed neurocognitive deficits, as most were unexplained by neuroanatomical variations.

We observed a negative association between amygdala volume and the EF component of monitoring measured by parental responses on the BRIEF. Monitoring is an EF skill reflecting an individual’s ability to self-regulate and adjust for task performance.^54^ Patients with higher amygdala volume displayed challenges with monitoring skills more than patients with lower amygdala volume. This finding is unreported in CKD literature and is interesting in the context of accelerated age-related growth of amygdala volume in the CKD group. The amygdala plays a key role in evaluating the saliency of information,^55^ which is in keeping with our finding of monitoring skills. Nonuniform, increased growth rates within the brain do not necessarily mean improved brain function^56,57^ and may contribute to emotional and social regulation challenges.^58^

Existing data support a relationship between kidney function and cognition.^9,11^ Until our previous work,^25^ no data we know of exhibited an association between kidney function and brain volume. We again showed that lower kidney function was associated with lower cerebellar GM volume, replicating our previous observation from a smaller sample limited to male participants only.^25^ The small number of female participants in the CKD group prohibited exploration interactions between sex and group. Sex had limited association with regional volume after applying the power proportion adjustment (eTables 6 and 10 in Supplement 1). We found no associations between kidney function and amygdala or cerebellar WM volumes, nor between proteinuria, anemia, or acidosis and cerebellar GM morphometry.

### Limitations

The findings of this case-control study should be considered within limitations. The modest sample size of this exploratory study highlights the need for replication in larger studies. Given the sample size, analyses were limited to linear models. Caution is warranted in attributing certainty to the associations found due to the number of statistical tests performed in a modestly-sized sample. Second, the current sample included mostly White individuals (88%) and more male participants in the pediatric CKD group (63.6%), indicating a need to diversify recruitment to ensure study findings are representative of the pediatric CKD community. Third, exclusion of patients with CKD with glomerular disease limits generalizability. This exclusion was purposeful to minimize the noise induced by a very different cause of CKD and associated therapies with neurotoxic effects.^59^ Additionally, the prevalence of CAKUT is higher among preterm infants than in the general population. Prematurity has known consequences for neurodevelopment.^60^ Most premature participants in this sample were born at least at 34 weeks’ gestation and none were extremely premature. Prematurely born participants did not cluster at the extremes of cerebellum and amygdala distributions (eFigure 4 in Supplement 1). While homogeneity of the sample was prioritized, the complexity of CKD cause and disease management makes it difficult to account for all potential factors that influence neurodevelopment. Nearly a third of the CKD sample took medication to manage high blood pressure. Hypertension has been associated with negative neurocognitive outcomes^15,61^ but did not appear to be associated with key neurocognitive outcomes here (eFigure 5 in Supplement 1). Similarly, use of psychotropic medications was higher in the CKD sample than unaffected controls. Unadjusted analyses showed no difference in cerebellum GM volume based on psychotropic medication use (eFigure 6 in Supplement 1). A larger sample is required to explore associations between these clinical variables and MRI-based assessment of neurodevelopment in CKD.

Our findings support our conclusion that CKD is associated with abnormal brain growth; however, the observed brain abnormalities in children with CAKUT could stem from primary developmental issues, secondary effects of CKD, or both. Although the CKD sample had what are considered isolated congenital anomalies of the kidney and urinary tract, factors leading to abnormal kidney development could impact brain development.^62,63^ Several genes linked to developmental kidney disorders play crucial roles in early brain development. For example, prenatal cerebellar development is linked to homeobox genes like PAX-2 and signaling genes like Fibroblast Growth Factor 8,^41,64,65^ which are associated with early kidney development and CKD causes such as nephron development disorders, kidney agenesis, and vesicoureteral reflux.^66,67,68,69,70,71^ It is conceivable that the observed cerebellar features indicate a predisposition to lower GM, with declining kidney function in pediatric CKD acting as a second hit that worsens this decline with age.

## Conclusions

In this case-control study of neurodevelopment in pediatric CKD, we explored age-related neurodevelopmental trends, providing evidence for abnormal growth trajectories of the cerebellum in this population. Our findings suggested that reduced cerebellum volume was associated with both lower kidney function and EF deficits. This is the first study we know of in the CKD literature to explicitly characterize age-related neurodevelopmental changes associated with CKD. The mechanism behind these differences remains unclear, highlighting the need to assess cerebellar-cortical neurocircuitry to understand executive dysfunction in CKD using novel models and study designs to distinguish the role of kidney pathophysiology from developmental abnormalities as predisposing factors for abnormal neurodevelopment in pediatric CKD.

## Notes

Supplement 1. **eTable 1. **Comparison of participants and nonparticipants **eTable 2. **Standardized neurocognitive assessment performed as part of study procedures **eTable 3. **Estimates for intracranial volume (ICV) association with regional volume (ROI) following the power proportion **eTable 4. **Exploring the impact of socioeconomic status on brain outcomes **eTable 5.** Estimated mean differences between groups on performance-based measures and parental surveys adjusted for SES **eTable 6.** Summary statistics for the age × group interaction with estimates for model association with scaled, power-proportion-adjusted ROIs **eTable 7. **Estimates for model association with scaled, power-proportion-adjusted cerebellar regions **eTable 8. **Univariate association analyses between neurocognitive outcomes and neuroanatomical volumes in patients with CKD **eTable 9. **Univariate association analyses between neurocognitive outcomes and the superior posterior lobe of the cerebellum in patients with CKD **eTable 10. **Estimate for the variable sex on regions of interest following adjustment for intracranial volume using the power proportion **eFigure 1. **Consort diagram for enrollment of patients with CKD **eFigure 2. **Age-related changes across regions of interest in control and patients with CKD **eFigure 3. **Age-related changes (x-axes) across cerebellum lobes in control (pink) and patients with CKD **eFigure 4. **Distribution of premature and term born participants across groups for regional volumes for which significant group differences were observed **eFigure 5. **Distribution of patients on blood pressure medication and those not on blood pressure medication for cerebellum gray matter volume **eFigure 6. **Distribution of participants on anxiety or depression medication in the sample

Supplement 2.
Data Sharing Statement

## References

[1] North American Pediatric Renal Trials and Collaborative Studies. Annual transplant report. 2014. Accessed December 23, 2024. https://naprtcs.org/system/files/2014_Annual_Transplant_Report.pdf

[2] Seikaly MG, Ho PL, Emmett L, Fine RN, Tejani A. Chronic renal insufficiency in children: the 2001 annual report of the NAPRTCS. Pediatr Nephrol. 2003;18(8):796-804. doi:10.1007/s00467-003-1158-5 12811650

[3] Giedd JN, Blumenthal J, Jeffries NO, . Brain development during childhood and adolescence: a longitudinal MRI study. Nat Neurosci. 1999;2(10):861-863. doi:10.1038/13158 10491603

[4] Gogtay N, Giedd JN, Lusk L, . Dynamic mapping of human cortical development during childhood through early adulthood. Proc Natl Acad Sci U S A. 2004;101(21):8174-8179. doi:10.1073/pnas.0402680101 15148381 PMC419576

[5] Tiemeier H, Lenroot RK, Greenstein DK, Tran L, Pierson R, Giedd JN. Cerebellum development during childhood and adolescence: a longitudinal morphometric MRI study. Neuroimage. 2010;49(1):63-70. doi:10.1016/j.neuroimage.2009.08.016 19683586 PMC2775156

[6] Lullmann O, Conrad AL, Steinbach EJ, Wilgenbusch T, Harshman LA, van der Plas E. Neurocognitive deficits may not resolve following pediatric kidney transplantation. Pediatr Transplant. 2023;27(4):e14505. doi:10.1111/petr.14505 36932049 PMC11001201

[7] Lullmann O, van der Plas E, Harshman LA. Understanding the impact of pediatric kidney transplantation on cognition: a review of the literature. Pediatr Transplant. 2023;27(8):e14597. doi:10.1111/petr.14597 37664967 PMC11034761

[8] Mendley SR, Matheson MB, Shinnar S, . Duration of chronic kidney disease reduces attention and executive function in pediatric patients. Kidney Int. 2015;87(4):800-806. doi:10.1038/ki.2014.323 25252026 PMC4372504

[9] Hooper SR, Gerson AC, Butler RW, . Neurocognitive functioning of children and adolescents with mild-to-moderate chronic kidney disease. Clin J Am Soc Nephrol. 2011;6(8):1824-1830. doi:10.2215/CJN.09751110 21737850 PMC3156421

[10] Gerson AC, Butler R, Moxey-Mims M, . Neurocognitive outcomes in children with chronic kidney disease: current findings and contemporary endeavors. Ment Retard Dev Disabil Res Rev. 2006;12(3):208-215. doi:10.1002/mrdd.20116 17061289

[11] Hooper SR, Gerson AC, Johnson RJ, . Neurocognitive, social-behavioral, and adaptive functioning in preschool children with mild to moderate kidney disease. J Dev Behav Pediatr. 2016;37(3):231-238. doi:10.1097/DBP.0000000000000267 26890559 PMC4818179

[12] Hooper SR, Laney N, Radcliffe J, . Executive functioning in children, adolescents, and young adults with chronic kidney disease. J Dev Behav Pediatr. 2015;36(9):734-742. doi:10.1097/DBP.0000000000000221 26468938

[13] Dobre M, Gaussoin SA, Bates JT, ; SPRINT Research Group. Serum bicarbonate concentration and cognitive function in hypertensive adults. Clin J Am Soc Nephrol. 2018;13(4):596-603. doi:10.2215/CJN.07050717 29567858 PMC5968905

[14] Harshman LA, Kogon AJ, Matheson MB, . Bicarbonate, blood pressure, and executive function in pediatric CKD-is there a link? Pediatr Nephrol. 2020;35(7):1323-1330. doi:10.1007/s00467-020-04507-5 32297000 PMC8077226

[15] Lande MB, Mendley SR, Matheson MB, . Association of blood pressure variability and neurocognition in children with chronic kidney disease. Pediatr Nephrol. 2016;31(11):2137-2144. doi:10.1007/s00467-016-3425-2 27263021 PMC5042825

[16] Tervo-Clemmens B, Calabro FJ, Parr AC, Fedor J, Foran W, Luna B. A canonical trajectory of executive function maturation from adolescence to adulthood. Nat Commun. 2023;14(1):6922. doi:10.1038/s41467-023-42540-8 37903830 PMC10616171

[17] Pomponio R, Erus G, Habes M, . Harmonization of large MRI datasets for the analysis of brain imaging patterns throughout the lifespan. Neuroimage. 2020;208:116450. doi:10.1016/j.neuroimage.2019.116450 31821869 PMC6980790

[18] Fair DA, Cohen AL, Power JD, . Functional brain networks develop from a “local to distributed” organization. PLoS Comput Biol. 2009;5(5):e1000381. doi:10.1371/journal.pcbi.1000381 19412534 PMC2671306

[19] Pines A, Keller AS, Larsen B, . Development of top-down cortical propagations in youth. Neuron. 2023;111(8):1316-1330.e5. doi:10.1016/j.neuron.2023.01.014 36803653 PMC10121821

[20] Schmahmann JD, Guell X, Stoodley CJ, Halko MA. The theory and neuroscience of cerebellar cognition. Ann Rev Neurosci. 2019;42:337-364. doi:10.1146/annurev-neuro-070918-050258 30939101

[21] Guell X, Gabrieli JDE, Schmahmann JD. Triple representation of language, working memory, social and emotion processing in the cerebellum: convergent evidence from task and seed-based resting-state fMRI analyses in a single large cohort. Neuroimage. 2018;172:437-449. doi:10.1016/j.neuroimage.2018.01.082 29408539 PMC5910233

[22] Koziol LF, Budding D, Andreasen N, . Consensus paper: the cerebellum’s role in movement and cognition. Cerebellum. 2014;13(1):151-177. doi:10.1007/s12311-013-0511-x 23996631 PMC4089997

[23] Clark SV, Semmel ES, Aleksonis HA, Steinberg SN, King TZ. Cerebellar-subcortical-cortical systems as modulators of cognitive functions. Neuropsychol Rev. 2021;31(3):422-446. doi:10.1007/s11065-020-09465-1 33515170

[24] Khalil M, Hollander P, Raucher-Chéné D, Lepage M, Lavigne KM. Structural brain correlates of cognitive function in schizophrenia: a meta-analysis. Neurosci Biobehav Rev. 2022;132:37-49. doi:10.1016/j.neubiorev.2021.11.034 34822878

[25] Solomon MA, van der Plas E, Langbehn KE, . Early pediatric chronic kidney disease is associated with brain volumetric gray matter abnormalities. Pediatr Res. 2021;89(3):526-532. doi:10.1038/s41390-020-01203-w 33069166 PMC7981243

[26] Hartung EA, Erus G, Jawad AF, . Brain magnetic resonance imaging findings in children and young adults with CKD. Am J Kidney Dis. 2018;72(3):349-359. doi:10.1053/j.ajkd.2017.11.024 29398180 PMC6070426

[27] Hollingshead AB. Four factor index of social status. 1975. Accessed December 23, 2024. https://artlesstanzim.wordpress.com/wp-content/uploads/2014/05/hollinghead-four-factors-2.pdf

[28] Gioia GA, Isquith PK, Kenworthy L. Behavior Rating Inventory of Executive Functions (BRIEF). Harcourt; 2000.

[29] Pierce CB, Muñoz A, Ng DK, Warady BA, Furth SL, Schwartz GJ. Age- and sex-dependent clinical equations to estimate glomerular filtration rates in children and young adults with chronic kidney disease. Kidney Int. 2021;99(4):948-956. doi:10.1016/j.kint.2020.10.047 33301749 PMC9083470

[30] White N, Roddey C, Shankaranarayanan A, . PROMO: Real-time prospective motion correction in MRI using image-based tracking. Magn Reson Med. 2010;63(1):91-105. doi:10.1002/mrm.22176 20027635 PMC2892665

[31] Pierson R, Johnson H, Harris G, . Fully automated analysis using BRAINS: AutoWorkup. Neuroimage. 2011;54(1):328-336. doi:10.1016/j.neuroimage.2010.06.047 20600977 PMC3827877

[32] Wang H, Suh JW, Das SR, Pluta JB, Craige C, Yushkevich PA. Multi-atlas segmentation with joint label fusion. IEEE Trans Pattern Anal Mach Intell. 2013;35(3):611-623. doi:10.1109/TPAMI.2012.143 22732662 PMC3864549

[33] Desikan RS, Ségonne F, Fischl B, . An automated labeling system for subdividing the human cerebral cortex on MRI scans into gyral based regions of interest. Neuroimage. 2006;31(3):968-980. doi:10.1016/j.neuroimage.2006.01.021 16530430

[34] Diedrichsen J, Balsters JH, Flavell J, Cussans E, Ramnani N. A probabilistic MR atlas of the human cerebellum. Neuroimage. 2009;46(1):39-46. doi:10.1016/j.neuroimage.2009.01.045 19457380

[35] Liu D, Johnson HJ, Long JD, Magnotta VA, Paulsen JS. The power-proportion method for intracranial volume correction in volumetric imaging analysis. Front Neurosci. 2014;8:356. doi:10.3389/fnins.2014.00356 25414635 PMC4222222

[36] Lenroot RK, Giedd JN. Sex differences in the adolescent brain. Brain Cogn. 2010;72(1):46-55. doi:10.1016/j.bandc.2009.10.008 19913969 PMC2818549

[37] Lenroot RK, Gogtay N, Greenstein DK, . Sexual dimorphism of brain developmental trajectories during childhood and adolescence. Neuroimage. 2007;36(4):1065-1073. doi:10.1016/j.neuroimage.2007.03.053 17513132 PMC2040300

[38] Fadrowski JJ, Neu AM, Schwartz GJ, Furth SL. Pediatric GFR estimating equations applied to adolescents in the general population. Clin J Am Soc Nephrol. 2011;6(6):1427-1435. doi:10.2215/CJN.06460710 21566103 PMC3109941

[39] van der Plas E, Solomon MA, Hopkins L, . Global and regional white matter fractional anisotropy in children with chronic kidney disease. J Pediatr. 2021;242:166-173.e3. 34758354 10.1016/j.jpeds.2021.11.006 PMC8882141

[40] Lijdsman S, Königs M, van Sandwijk MS, . Structural brain abnormalities in children and young adults with severe chronic kidney disease. Pediatr Nephrol. 2021;37(5):1125-1136. 34800137 10.1007/s00467-021-05276-5 PMC9023396

[41] Wang VY, Zoghbi HY. Genetic regulation of cerebellar development. Nat Rev Neurosci. 2001;2(7):484-491. doi:10.1038/35081558 11433373

[42] Wang SS, Kloth AD, Badura A. The cerebellum, sensitive periods, and autism. Neuron. 2014;83(3):518-532. doi:10.1016/j.neuron.2014.07.016 25102558 PMC4135479

[43] Gaiser C, van der Vliet R, de Boer AAA, . Population-wide cerebellar growth models of children and adolescents. Nat Commun. 2024;15(1):2351. doi:10.1038/s41467-024-46398-2 38499518 PMC10948906

[44] Stoodley CJ. The cerebellum and neurodevelopmental disorders. Cerebellum. 2016;15(1):34-37. doi:10.1007/s12311-015-0715-3 26298473 PMC4811332

[45] Wierenga L, Langen M, Ambrosino S, van Dijk S, Oranje B, Durston S. Typical development of basal ganglia, hippocampus, amygdala and cerebellum from age 7 to 24. Neuroimage. 2014;96:67-72. doi:10.1016/j.neuroimage.2014.03.072 24705201

[46] Beuriat PA, Cristofori I, Gordon B, Grafman J. The shifting role of the cerebellum in executive, emotional and social processing across the lifespan. Behav Brain Funct. 2022;18(1):6. doi:10.1186/s12993-022-00193-5 35484543 PMC9047369

[47] Horton AM Jr, Soper HV, Reynolds CR. Executive functions in children with traumatic brain injury. Appl Neuropsychol. 2010;17(2):99-103. doi:10.1080/09084281003708944 20467949

[48] Limperopoulos C, du Plessis AJ. Disorders of cerebellar growth and development. Curr Opin Pediatr. 2006;18(6):621-627. doi:10.1097/MOP.0b013e32801080e8 17099360

[49] Schmahmann J, Pandya D. Fiber pathways of the brain. OUP; 2009.

[50] Schmahmann JD. Disorders of the cerebellum: ataxia, dysmetria of thought, and the cerebellar cognitive affective syndrome. J Neuropsychiatry Clin Neurosci. 2004;16(3):367-378. doi:10.1176/jnp.16.3.367 15377747

[51] Schmahmann JD, Sherman JC. The cerebellar cognitive affective syndrome. Brain. 1998;121(Pt 4):561-579. doi:10.1093/brain/121.4.561 9577385

[52] Schmahmann JD, Guell X, Stoodley CJ, Halko MA. The theory and neuroscience of cerebellar cognition. Annu Rev Neurosci. 2019;42:337-364. doi:10.1146/annurev-neuro-070918-050258 30939101

[53] King M, Hernandez-Castillo CR, Poldrack RA, Ivry RB, Diedrichsen J. Functional boundaries in the human cerebellum revealed by a multi-domain task battery. Nat Neurosci. 2019;22(8):1371-1378. doi:10.1038/s41593-019-0436-x 31285616 PMC8312478

[54] Ridderinkhof KR, van den Wildenberg WP, Segalowitz SJ, Carter CS. Neurocognitive mechanisms of cognitive control: the role of prefrontal cortex in action selection, response inhibition, performance monitoring, and reward-based learning. Brain Cogn. 2004;56(2):129-140. doi:10.1016/j.bandc.2004.09.016 15518930

[55] Cunningham WA, Brosch T. Motivational salience:amygdala tuning from traits, needs, values, and goals. Curr Dir Psychol Sci. 2012;21(1):54-59. doi:10.1177/0963721411430832

[56] Piven J, Elison JT, Zylka MJ. Toward a conceptual framework for early brain and behavior development in autism. Mol Psychiatry. 2017;22(10):1385-1394. doi:10.1038/mp.2017.131 28937691 PMC5621737

[57] Gandy K, Koscik TR, Alexander T, Steinberg JD, Krull KR, van der Plas E. Characterization of brain development with neuroimaging in a female mouse model of chemotherapy treatment of acute lymphoblastic leukemia. Transl Pediatr. 2024;13(3):408-416. doi:10.21037/tp-23-458 38590373 PMC10998997

[58] Goodwill AM, Low LT, Fox PT, . Meta-analytic connectivity modelling of functional magnetic resonance imaging studies in autism spectrum disorders. Brain Imaging Behav. 2023;17(2):257-269. doi:10.1007/s11682-022-00754-2 36633738 PMC10049951

[59] Bechstein WO. Neurotoxicity of calcineurin inhibitors: impact and clinical management. Transpl Int. 2000;13(5):313-326. doi:10.1111/j.1432-2277.2000.tb01004.x 11052266

[60] Ortinau C, Neil J. The neuroanatomy of prematurity: normal brain development and the impact of preterm birth. Clin Anat. 2015;28(2):168-183. doi:10.1002/ca.22430 25043926

[61] Lande MB, Gerson AC, Hooper SR, . Casual blood pressure and neurocognitive function in children with chronic kidney disease: a report of the children with chronic kidney disease cohort study. Clin J Am Soc Nephrol. 2011;6(8):1831-1837. doi:10.2215/CJN.00810111 21700829 PMC3156422

[62] Verbitsky M, Kogon AJ, Matheson M, . Genomic disorders and neurocognitive impairment in pediatric CKD. J Am Soc Nephrol. 2017;28(8):2303-2309. doi:10.1681/ASN.2016101108 28348065 PMC5533237

[63] Kolvenbach CM, Shril S, Hildebrandt F. The genetics and pathogenesis of CAKUT. Nat Rev Nephrol. 2023;19(11):709-720. doi:10.1038/s41581-023-00742-9 37524861

[64] Namm A, Arend A, Aunapuu M. Expression of Pax2 protein during the formation of the central nervous system in human embryos. Folia Morphol (Warsz). 2014;73(3):272-278. doi:10.5603/FM.2014.0043 25346342

[65] Suzuki A, Harada H, Nakamura H. Nuclear translocation of FGF8 and its implication to induce Sprouty2. Dev Growth Differ. 2012;54(4):463-473. doi:10.1111/j.1440-169X.2012.01332.x 22404534

[66] Dziarmaga A, Quinlan J, Goodyer P. Renal hypoplasia: lessons from Pax2. Pediatr Nephrol. 2006;21(1):26-31. doi:10.1007/s00467-005-2039-x 16273412

[67] Martinovic-Bouriel J, Benachi A, Bonnière M, . PAX2 mutations in fetal renal hypodysplasia. Am J Med Genet A. 2010;152A(4):830-835. doi:10.1002/ajmg.a.33133 20358591

[68] Zheng Y, Xu J, Guo W, . The significance of Pax2 expression in the ureter epithelium of children with vesicoureteric reflux. Hum Pathol. 2015;46(7):963-970. doi:10.1016/j.humpath.2015.01.007 25912758

[69] de Miranda DM, Dos Santos Júnior AC, Dos Reis GS, . PAX2 polymorphisms and congenital abnormalities of the kidney and urinary tract in a Brazilian pediatric population: evidence for a role in vesicoureteral reflux. Mol Diagn Ther. 2014;18(4):451-457. doi:10.1007/s40291-014-0096-1 24633556

[70] Boualia SK, Gaitan Y, Murawski I, Nadon R, Gupta IR, Bouchard M. Vesicoureteral reflux and other urinary tract malformations in mice compound heterozygous for Pax2 and Emx2. PLoS One. 2011;6(6):e21529. doi:10.1371/journal.pone.0021529 21731775 PMC3123351

[71] Bates CM. Role of fibroblast growth factor receptor signaling in kidney development. Am J Physiol Renal Physiol. 2011;301(2):F245-F251. doi:10.1152/ajprenal.00186.2011 21613421 PMC3154593

