# Supplementary material for: Age-Related Changes in Brain Structure in Pediatric Chronic Kidney Disease
Source: JAMA Netw Open. 2025 Feb 3;8(2):e2457601. doi: 10.1001/jamanetworkopen.2024.57601 (PMC11791706; doi:10.1001/jamanetworkopen.2024.57601)
Supplement: Supplement 1. — eTable 1. Comparison of participants and nonparticipants eTable 2. Standardized neurocognitive assessment performed as part of study procedures eTable 3. Estimates for intracranial volume (ICV) association with regional volume (ROI) following the power proportion eTable 4. Exploring the impact of socioeconomic status on brain outcomes eTable 5. Estimated mean differences between groups on performance-based measures and parental surveys adjusted for SES eTable 6. Summary statistics for the age × group interaction with estimates for model association with scaled, power-proportion-adjusted ROIs eTable 7. Estimates for model association with scaled, power-proportion-adjusted cerebellar regions eTable 8. Univariate association analyses between neurocognitive outcomes and neuroanatomical volumes in patients with CKD eTable 9. Univariate association analyses between neurocognitive outcomes and the superior posterior lobe of the cerebellum in patients with CKD eTable 10. Estimate for the variable sex on regions of interest following adjustment for intracranial volume using the power proportion eFigure 1. Consort diagram for enrollment of patients with CKD eFigure 2. Age-related changes across regions of interest in control and patients with CKD eFigure 3. Age-related changes (x-axes) across cerebellum lobes in control (pink) and patients with CKD eFigure 4. Distribution of premature and term born participants across groups for regional volumes for which significant group differences were observed eFigure 5. Distribution of patients on blood pressure medication and those not on blood pressure medication for cerebellum gray matter volume eFigure 6. Distribution of participants on anxiety or depression medication in the sample [file jamanetwopen-e2457601-s001.pdf]

Supplemental Online Content

van der Plas E, Nelson E, Becknell B, et al. Age-related changes in brain structure in pediatric chronic kidney disease. *JAMA Netw Open*. 2025;8(2):e2457601.
doi:10.1001/jamanetworkopen.2024.57601

eTable 1. Comparison of participants and nonparticipants

eTable 2. Standardized neurocognitive assessment performed as part of study procedures

eTable 3. Estimates for intracranial volume (ICV) association with regional volume (ROI) following the power proportion

eTable 4. Exploring the impact of socioeconomic status on brain outcomes

eTable 5. Estimated mean differences between groups on performance-based measures and parental surveys adjusted for SES

eTable 6. Summary statistics for the age × group interaction with estimates for model association with scaled, power-proportion-adjusted ROIs

eTable 7. Estimates for model association with scaled, power-proportion-adjusted cerebellar regions

eTable 8. Univariate association analyses between neurocognitive outcomes and neuroanatomical volumes in patients with CKD

eTable 9. Univariate association analyses between neurocognitive outcomes and the superior posterior lobe of the cerebellum in patients with CKD

© 2024 van der Plas E et al. *JAMA Network Open*.

eTable 10. Estimate for the variable sex on regions of interest following adjustment for intracranial volume using the power proportion

eFigure 1. Consort diagram for patient enrollment for patients with CKD

eFigure 2. Age-related changes across regions of interest in control and patients with CKD

eFigure 3. Age-related changes (x-axes) across cerebellum lobes in control (pink) and patients with CKD

eFigure 4. Distribution of premature and term born participants across groups for regional volumes for which significant group differences were observed

eFigure 5. Distribution of patients on blood pressure medication and those not on blood pressure medication for cerebellum gray matter volume

eFigure 6. Distribution of participants on anxiety or depression medication in the sample

This supplemental material has been provided by the authors to give readers additional information about their work.

© 2024 van der Plas E et al. *JAMA Network Open*.

eTable 1. Comparison of participants and nonparticipants.

Compared to participants, non-participants were ~4 years older (p<0.001); but the groups did not differ on other relevant variables, including sex, race, ethnicity, and CKD Stage.

	**Participants (N=37)**	**Non-Participants (N=33)**	**p-value**
**Age (yrs)**			
Mean (SD)	12.4 (4.05)	16.8 (4.92)	<0.001
Median [Min-Max]	12.7 [6.25-21.8]	17.9 [6.55-24.8]	
**Sex**			
Females	7 (18.9%)	8 (24.2%)	0.80
Males	30 (81.1%)	25 (75.8%)	
**CKD Stage**			
Stage 1	9 (24.3%)	11 (33.3%)	0.48
Stage 2	16 (43.2%)	16 (48.5%)	
Stage 3	11 (29.7%)	6 (18.2%)	
Stage 4	1 (2.7%)	0 (0%)	
**Race**			
White	32 (86.5%)	29 (87.9%)	0.39
Black	1 (2.7%)	1 (3.0%)	
Multiracial	1 (2.7%)	3 (9.1%)	
Asian American	2 (5.4%)	0 (0%)	
Missing	1 (2.7%)	0 (0%)	
**Ethnicity**			
Not Hispanic/Latino	33 (89.2%)	31 (93.9%)	0.78
Hispanic/Latino	4 (10.8%)	2 (6.1%)	

© 2024 van der Plas E et al. *JAMA Network Open*.

eTable 2. Standardized neurocognitive assessment performed as part of study procedures.

Child-Administered Tests	Domain
Cognition (WISC-IV / WAIS-IV)	
Similarities	Verbal Comprehension
Vocabulary	Verbal Comprehension
Block Design	Perceptual Reasoning
Matrix Reasoning	Perceptual Reasoning
Digit-Symbol Coding	Processing Speed
Symbol Search	Processing Speed
Digit Span	Working Memory
Spatial Span	Working Memory
Executive Function (DKEFS)	
Verbal Fluency	Initiation
Color/Word Interference	Switching
Category Sorting	Sorting
Academic Achievement (WRAT-IV)	
Arithmetic	Numerical Computation
Reading	Word Identification
Motor Dexterity	
Grooved Pegboard	Fine Motor, Motor Speed
Child-Report Questionnaires	
Self-Description Questionnaire (SDQ I, II, III)	Self-Concept
**Proxy-Report Questionnaires**	**Domain**
Family History	
Birth History	
BRIEF Parent/Informant	Executive Function

Notes. Wechsler Intelligence Scale for Children, 4th Edition (WISC-IV; child participants). Wechsler Adult Intelligence Scale, 4th Edition (WAIS-IV; adult participants). Delis-Kaplan Executive Function System (DKEFS). Wide Range Achievement Test, 4th Edition (WRAT-4). SDQ I (ages 7-12); SDQ II (ages 13-17); SDQ III (18+). Behavior Rating Inventory of Executive Function, 2nd Edition (BRIEF-2; Parent Report for child participants and Proxy Report for adult participants).

© 2024 van der Plas E et al. *JAMA Network Open*.

eTable 3. Estimates for intracranial volume (ICV) association with regional volume (ROI) following the power proportion*. Neuroanatomical regions of interest (ROIs) vary with intracranial volume (ICV). Use of the power proportion method resulted in effective de-trending of the relationship between ROI and ICV.

ROI	*Estimate for ICV (SD)*	*p*
Cerebral GM	-0.00000020 (0.00000072)	0.78
Cerebral WM	0.00000093 (0.00000070)	0.19
Cerebellar GM	-0.00000008 (0.00000072)	0.91
Cerebellar WM	0.00000077 (0.00000071)	0.28
Frontal GM	0.00000003 (0.00000072)	0.97
Frontal WM	0.00000104 (0.00000070)	0.14
Parietal GM	-0.00000024 (0.00000072)	0.74
Parietal WM	0.00000069 (0.00000071)	0.34
Temporal GM	-0.00000038 (0.00000072)	0.59
Temporal WM	0.00000049 (0.00000072)	0.50
Occipital GM	-0.00000031 (0.00000072)	0.67
Occipital WM	0.00000007 (0.00000072)	0.92
Basal Ganglia	0.00000024 (0.00000072)	0.74
Caudate	0.00000083 (0.00000071)	0.25
Putamen	-0.00000059 (0.00000071)	0.41
Thalamus	0.00000080 (0.00000072)	0.27
Hippocampus	0.00000067 (0.00000072)	0.35
Amygdala	0.00000072 (0.00000071)	0.31

*Relationships between ROIs and ICV follow the power law principle, i.e., $ROI = \alpha ICV^{\beta}$, where α is the constant and β represents the scaling component of the power function. For each ROI, a non-linear model was fit to determine the value of α and β. The power proportion (PPM) was subsequently calculated by dividing the ROI volume by the ICV^{β} (i.e., $ROI_{ppm}=\frac{ROI}{ICV^{\beta}}$).

© 2024 van der Plas E et al. *JAMA Network Open*.

eTable 4. Exploring the impact of socioeconomic status on brain outcomes.

Dependent Var	*Independent Var*	*Contrast*	*Estimate*	*STD.Error*	*p*
Cerebral GM**	Group	Controls vs. CKD	-0.01	0.180	0.954
	Sex	Females vs. Males	0.10	0.169	0.566
	SES	Prof. vs. semi/skilled*	-0.29	0.169	0.0903
	Age		-0.13	0.019	2.79e-10
Cerebral WM	Group	Controls vs. CKD	-0.19	0.144	0.2
	Sex	Females vs. Males	-0.22	0.135	0.108
	SES	Prof. vs. semi/skilled*	-0.09	0.135	0.486
	Age		0.17	0.015	5.25e-20
Cerebellar GM	Group	Controls vs. CKD	-0.39	0.192	0.0465
	Sex	Females vs. Males	0.19	0.179	0.285
	SES	Prof. vs. semi/skilled*	0.23	0.180	0.199
	Age		-0.02	0.020	0.349
Cerebellar WM	Group	Controls vs. CKD	-0.37	0.205	0.0757
	Sex	Females vs. Males	-0.22	0.193	0.264
	SES	Prof. vs. semi/skilled*	0.26	0.193	0.186
	Age		0.08	0.022	0.000464
Frontal GM	Group	Controls vs. CKD	-0.02	0.188	0.929
	Sex	Females vs. Males	-0.08	0.177	0.655
	SES	Prof. vs. semi/skilled*	-0.03	0.176	0.874
	Age		-0.13	0.020	1.96e-09
Frontal WM	Group	Controls vs. CKD	-0.25	0.146	0.0912
	Sex	Females vs. Males	-0.30	0.137	0.0294
	SES	Prof. vs. semi/skilled*	0.06	0.137	0.64
	Age		0.17	0.015	1.3e-18
Parietal GM	Group	Controls vs. CKD	-0.04	0.176	0.807
	Sex	Females vs. Males	0.10	0.165	0.558
	SES	Prof. vs. semi/skilled*	-0.32	0.165	0.0548
	Age		-0.13	0.019	2.43e-10
Parietal WM	Group	Controls vs. CKD	-0.12	0.159	0.434
	Sex	Females vs. Males	-0.13	0.149	0.4
	SES	Prof. vs. semi/skilled*	-0.15	0.149	0.331
	Age		0.17	0.017	9.47e-17
Temporal GM	Group	Controls vs. CKD	-0.11	0.215	0.607
	Sex	Females vs. Males	0.31	0.202	0.124
	SES	Prof. vs. semi/skilled*	-0.28	0.201	0.17
	Age		-0.05	0.023	0.0212
Temporal WM	Group	Controls vs. CKD	-0.20	0.182	0.267
	Sex	Females vs. Males	-0.03	0.171	0.845
	SES	Prof. vs. semi/skilled*	-0.22	0.170	0.19
	Age		0.13	0.019	8.4e-10
Occipital GM	Group	Controls vs. CKD	0.29	0.204	0.159
	Sex	Females vs. Males	0.09	0.191	0.65
	SES	Prof. vs. semi/skilled*	-0.46	0.191	0.0188
	Age		-0.08	0.022	0.00038
Occipital WM	Group	Controls vs. CKD	0.27	0.214	0.212
	Sex	Females vs. Males	0.04	0.201	0.824
	SES	Prof. vs. semi/skilled*	-0.49	0.201	0.0155
	Age		0.03	0.023	0.132
Basal Ganglia	Group	Controls vs. CKD	-0.16	0.220	0.456
	Sex	Females vs. Males	0.08	0.207	0.692
	SES	Prof. vs. semi/skilled*	-0.08	0.207	0.686

© 2024 van der Plas E et al. *JAMA Network Open*.

	Age		-0.01	0.023	0.692
Caudate	Group	Controls vs. CKD	-0.20	0.220	0.359
	Sex	Females vs. Males	-0.12	0.206	0.549
	SES	Prof. vs. semi/skilled*	-0.15	0.206	0.477
	Age		0.02	0.023	0.322
Putamen	Group	Controls vs. CKD	0.05	0.219	0.825
	Sex	Females vs. Males	0.26	0.206	0.203
	SES	Prof. vs. semi/skilled*	0.12	0.205	0.546
	Age		-0.03	0.023	0.137
Thalamus	Group	Controls vs. CKD	-0.49	0.218	0.0264
	Sex	Females vs. Males	-0.04	0.205	0.859
	SES	Prof. vs. semi/skilled*	0.05	0.205	0.792
	Age		0.03	0.023	0.159
Hippocampus	Group	Controls vs. CKD	-0.19	0.190	0.315
	Sex	Females vs. Males	0.00	0.178	0.989
	SES	Prof. vs. semi/skilled*	-0.09	0.178	0.617
	Age		0.09	0.020	4.61e-05
Amygdala	Group	Controls vs. CKD	-0.03	0.209	0.87
	Sex	Females vs. Males	-0.18	0.197	0.37
	SES	Prof. vs. semi/skilled*	-0.16	0.196	0.412
	Age		0.07	0.022	0.00102

*Professional/high managerial positions vs. semi skilled/skilled workers' **WM=White Matter; GM=Gray Matter

© 2024 van der Plas E et al. *JAMA Network Open*.

eTable 5. Estimated mean differences between groups on performance-based measures and parental surveys adjusted for SES.

Variable	*Estimated Mean Difference*	*95% CI*	*p*
Pegboard Dominant Hand	-0.84	-1.34:-0.35	0.001
Verbal Comprehension Index	-0.28	-0.64:0.08	0.13
Perceptual Reasoning Index	-0.53	-0.92:-0.14	0.008
Processing Speed Index	-0.41	-0.77:-0.05	0.02
General Ability Index	-0.46	-0.81:-0.11	0.01
DKEFS Letter Fluency	-0.28	-0.69:0.13	0.18
DKEFS Category Fluency	-0.20	-0.67:0.27	0.40
DKEFS Category Switching	-0.56	-0.99:-0.13	0.01
DKEFS Color-Word Inference	-0.42	-0.73:-0.10	0.009
DKEFS Confirmed Correct Sorting	-0.61	-0.97:-0.24	0.001
DKEFS Free Sorting	-0.56	-0.91:-0.21	0.002
WRAT4 Reading	-0.37	-0.75:0.01	0.06
WRAT4 Arithmetic	-0.24	-0.63:0.15	0.23
BRIEF Inhibit	-0.41	-0.77:-0.05	0.03
BRIEF Shift	-0.27	-0.70:0.16	0.21
BRIEF Emotional Control	-0.74	-1.14:-0.34	< 0.001
BRIEF Initiate	-0.42	-0.79:-0.04	0.03
BRIEF Working Memory	-0.86	-1.23:-0.48	< 0.001
BRIEF Plan/Organize	-0.57	-0.96:-0.19	0.004
BRIEF Organization of Materials	-0.53	-0.93:-0.13	0.01
BRIEF Monitor	-0.65	-1.07:-0.24	0.002

© 2024 van der Plas E et al. *JAMA Network Open*.

eTable 6. Summary statistics for the age × group interaction with estimates for model association with scaled, power-proportion-adjusted ROIs.

ROI	*Predictor*	*Estimate*	*STE*	*p*
Cerebral GM	Group	0.30	0.57	0.60
	Age	-0.12	0.02	<0.001
	Sex	0.08	0.17	0.64
	Group*Age	-0.03	0.04	0.52
Cerebral WM	Group	-0.24	0.45	0.60
	Age	0.17	0.02	<0.001
	Sex	-0.21	0.14	0.12
	Group*Age	0.00	0.03	0.92
Cerebellar GM	Group	0.87	0.58	0.14
	Age	0.01	0.02	0.58
	Sex	0.17	0.17	0.34
	Group*Age	-0.10	0.04	0.03
Cerebellar WM	Group	0.88	0.63	0.16
	Age	0.10	0.02	<0.001
	Sex	-0.26	0.19	0.17
	Group*Age	-0.09	0.05	0.05
Frontal GM	Group	0.59	0.58	0.31
	Age	-0.11	0.02	<0.001
	Sex	-0.07	0.17	0.69
	Group*Age	-0.05	0.04	0.26
Frontal WM	Group	-0.06	0.46	0.89
	Age	0.17	0.02	<0.001
	Sex	-0.28	0.14	0.04
	Group*Age	-0.01	0.03	0.69
Parietal GM	Group	-0.05	0.56	0.93
	Age	-0.14	0.02	<0.001
	Sex	0.06	0.17	0.72
	Group*Age	-0.00	0.04	0.98
Parietal WM	Group	-0.69	0.50	0.17
	Age	0.14	0.02	<0.001
	Sex	-0.13	0.15	0.38
	Group*Age	0.04	0.04	0.24
Temporal GM	Group	-0.27	0.67	0.69
	Age	-0.05	0.02	0.05
	Sex	0.31	0.20	0.12
	Group*Age	0.01	0.05	0.87
Temporal WM	Group	-0.36	0.57	0.53
	Age	0.13	0.02	<0.001
	Sex	-0.02	0.17	0.92
	Group*Age	0.01	0.04	0.84
Occipital GM	Group	0.52	0.65	0.43
	Age	-0.07	0.02	0.005
	Sex	0.06	0.19	0.77
	Group*Age	-0.02	0.05	0.63
Occipital WM	Group	0.44	0.69	0.52
	Age	0.04	0.03	0.08
	Sex	0.02	0.20	0.93
	Group*Age	-0.02	0.05	0.67

© 2024 van der Plas E et al. *JAMA Network Open*.

Basal Ganglia	Group	0.46	0.69	0.51
	Age	-0.01	0.03	0.74
	Sex	-0.02	0.21	0.92
	Group*Age	-0.05	0.05	0.35
Caudate	Group	0.23	0.69	0.74
	Age	0.02	0.03	0.39
	Sex	-0.22	0.20	0.29
	Group*Age	-0.03	0.05	0.51
Putamen	Group	0.24	0.68	0.73
	Age	-0.04	0.03	0.15
	Sex	0.23	0.20	0.26
	Group*Age	-0.01	0.05	0.80
Thalamus	Group	-0.30	0.67	0.66
	Age	0.04	0.02	0.16
	Sex	-0.03	0.20	0.87
	Group*Age	-0.01	0.05	0.77
Hippocampus	Group	-0.61	0.63	0.34
	Age	0.09	0.02	<0.001
	Sex	-0.08	0.19	0.65
	Group*Age	0.03	0.05	0.53
Amygdala	Group	-1.19	0.64	0.07
	Age	0.05	0.02	0.03
	Sex	-0.17	0.19	0.38
	Group*Age	0.09	0.05	0.06

GM: Gray Matter. WM: White Matter

eTable 7. Estimates for models association with scaled, power-proportion-adjusted cerebellar regions

ROI	*Predictor*	*Estimate*	*STE*	*p*
Anterior Lobe	Group	0.63	0.68	0.36
	Age	0.06	0.03	0.04
	Sex	0.21	0.20	0.31
	Group*Age	-0.09	0.05	0.07
Superior Posterior Lobe	Group	0.73	0.68	0.29
	Age	0.03	0.03	0.28
	Sex	0.20	0.20	0.33
	Group*Age	-0.10	0.05	0.05
Inferior Posterior Lobe	Group	0.31	0.70	0.66
	Age	-0.00	0.03	0.93
	Sex	0.35	0.21	0.10
	Group*Age	-0.04	0.05	0.4
Flocculonodular Lobe	Group	1.08	0.67	0.11
	Age	0.10	0.03	<0.001
	Sex	-0.12	0.20	0.56
	Group*Age	-0.09	0.05	0.07
Vermis	Group	0.21	0.71	0.77
	Age	0.02	0.03	0.37
	Sex	0.07	0.21	0.74
	Group*Age	-0.02	0.05	0.78

© 2024 van der Plas E et al. *JAMA Network Open*.

eTable 8. Univariate association analyses between neurocognitive outcomes and neuroanatomical volumes in patients with CKD

Predictor	*Outcome*	*Estimate*	*STE*	*p*
Cerebellum GM	Pegboard Dominant Hand	0.227	0.351	0.52
	Perceptual Reasoning Index	0.031	0.218	0.89
	Processing Speed Index	0.145	0.257	0.58
	General Ability Index	0.009	0.205	0.97
	DKEFS Category Switching	-0.062	0.256	0.81
	DKEFS Color-Word Inference	0.008	0.230	0.97
	DKEFS Confirmed Correct Sorting	0.068	0.251	0.79
	DKEFS Free Sorting	0.024	0.237	0.92
	Inhibit	0.002	0.295	0.99
	Emotional Control	-0.204	0.285	0.48
	Initiate	0.143	0.308	0.61
	Working Memory	0.279	0.308	0.37
	**Plan/Organize**	**0.545**	**0.248**	**0.04**
	Organization of Materials	-0.305	0.304	0.33
	Monitor	0.101	0.286	0.71
Cerebellum WM	Pegboard Dominant Hand	0.250	0.278	0.38
	Perceptual Reasoning Index	0.087	0.177	0.61
	Processing Speed Index	0.333	0.199	0.11
	General Ability Index	0.227	0.164	0.17
	DKEFS Category Switching	0.196	0.219	0.38
	DKEFS Color-Word Inference	0.198	0.173	0.26
	DKEFS Confirmed Correct Sorting	0.370	0.202	0.08
	DKEFS Free Sorting	0.294	0.189	0.13
	Inhibit	0.368	0.201	0.08
	**Emotional Control**	**0.417**	**0.195**	**0.04**
	Initiate	-0.135	0.220	0.55
	Working Memory	0.178	0.222	0.44
	Plan/Organize	-0.015	0.196	0.94
	Organization of Materials	0.021	0.223	0.92
	Monitor	0.211	0.204	0.31
Amygdala	Pegboard Dominant Hand	0.307	0.249	0.23
	Perceptual Reasoning Index	-0.169	0.155	0.29
	Processing Speed Index	-0.358	0.184	0.06
	General Ability Index	-0.095	0.149	0.53
	DKEFS Category Switching	-0.084	0.220	0.71
	DKEFS Color-Word Inference	-0.025	0.176	0.89
	DKEFS Confirmed Correct Sorting	-0.006	0.212	0.98
	DKEFS Free Sorting	0.195	0.192	0.32
	Inhibit	-0.143	0.195	0.47
	Emotional Control	-0.361	0.182	0.06
	Initiate	-0.220	0.201	0.28
	Working Memory	0.060	0.208	0.78
	Plan/Organize	-0.113	0.180	0.54
	Organization of Materials	0.051	0.206	0.81
	**Monitor**	**-0.399**	**0.178**	**0.03**

Bolding denotes significance associations between the predictor and the outcome

© 2024 van der Plas E et al. *JAMA Network Open*.

eTable 9. Univariate association analyses between neurocognitive outcomes and the superior posterior lobe of the cerebellum in patients with CKD

Outcome	*Sup-Post Estimate*	*STE*	*p*
Pegboard Dominant Hand	0.358	0.324	0.28
Perceptual Reasoning Index	-0.024	0.195	0.90
Processing Speed Index	-0.012	0.207	0.95
General Ability Index	-0.010	0.184	0.96
DKEFS Category Switching	-0.206	0.223	0.37
DKEFS Color-Word Inference	-0.264	0.188	0.17
DKEFS Confirmed Correct Sorting	0.139	0.217	0.53
DKEFS Free Sorting	0.084	0.197	0.67
Inhibit	0.140	0.268	0.61
Emotional Control	-0.212	0.250	0.41
Initiate	-0.088	0.285	0.76
Working Memory	0.000	0.272	0.99
Plan/Organize	0.250	0.229	0.28
Organization of Materials	-0.428	0.271	0.13
Monitor	0.008	0.256	0.98

© 2024 van der Plas E et al. *JAMA Network Open*.

eTable 10. Estimate for the variable sex on regions of interest following adjustment for intracranial volume using the power proportion

ROI	*Estimate (SD)*	*p*
Cerebral GM	0.228 (0.225)	0.31
Cerebral WM	-0.568 (0.219)	0.01
Cerebellar GM	0.257 (0.197)	0.19
Cerebellar WM	-0.489 (0.221)	0.03
Frontal GM	0.057 (0.226)	0.80
Frontal WM	-0.654 (0.217)	0.003
Parietal GM	0.202 (0.225)	0.37
Parietal WM	-0.440 (0.222)	0.05
Temporal GM	0.364 (0.223)	0.11
Temporal WM	-0.287 (0.224)	0.20
Occipital GM	0.247 (0.225)	0.27
Occipital WM	0.033 (0.226)	0.88
Basal Ganglia	-0.059 (0.226)	0.80
Caudate	-0.400 (0.223)	0.08
Putamen	0.391 (0.223)	0.08
Thalamus	-0.317 (0.224)	0.16
Hippocampus	-0.357 (0.223)	0.11
Amygdala	-0.416 (0.222)	0.06

© 2024 van der Plas E et al. *JAMA Network Open*.

eFigure 1. Consort diagram for enrollment of patients with CKD .

Potentially eligible participants were identified using a query of the electronic medical record, searching for diagnosis codes specific to CKD (CKD stage 1, CKD stage 2, CKD stage 3, or CKD stage unspecified) and ages 6-21. Patients with diagnoses of kidney transplant or end stage kidney disease on chronic dialysis were excluded from the initial query. The EMR was used to confirm 1) accuracy of age and clinical diagnosis of CKD, 2) estimated glomerular filtration rate (eGFR) greater than 30 ml/min/1.73m^2 within 6 months of study participation using the CKiD U25 equation,[30] and 3) etiology of CKD due to CAKUT. Diagnoses representative of CAKUT included bilateral renal hypoplasia/dysplasia, posterior urethral valves, multi-cystic dysplastic kidney, bilateral moderate to severe vesicoureteral reflux and/or moderate to severe hydronephrosis associated with > 6 months of reduced kidney function. Solitary kidney diagnosis was not included unless there was a concomitant history of reduced kidney function (eGFR < 90 ml/min/1.73m^2 for more than 6 months) and other structural kidney abnormalities such as vesicoureteral reflux or renal hypoplasia/dysplasia.

Potentially Eligible
N=224

© 2024 van der Plas E et al. *JAMA Network Open*.

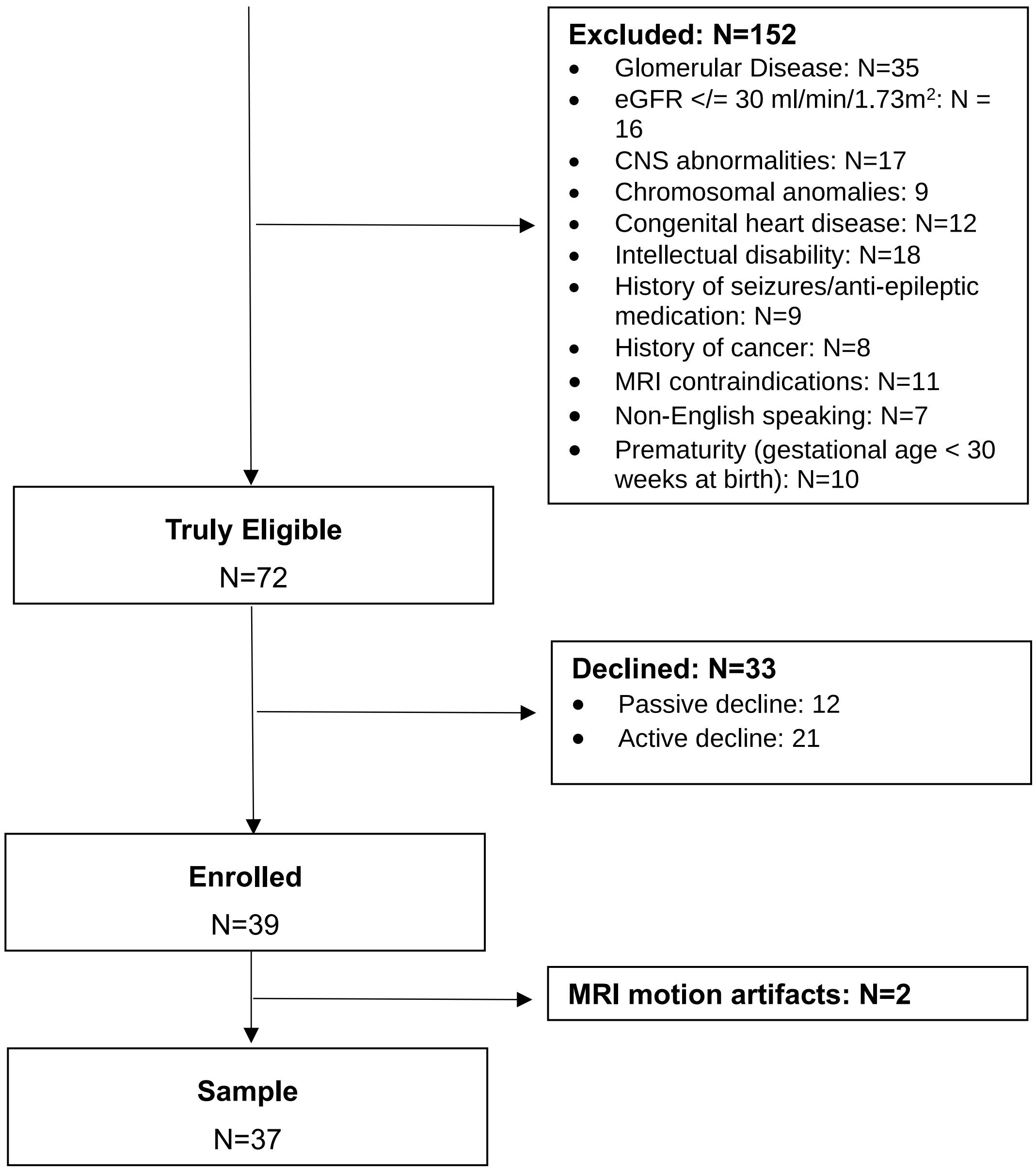

© 2024 van der Plas E et al. *JAMA Network Open*.

eFigure 2. Age-related changes (x-axes) across regions of interest in control (pink) and patients with CKD (blue

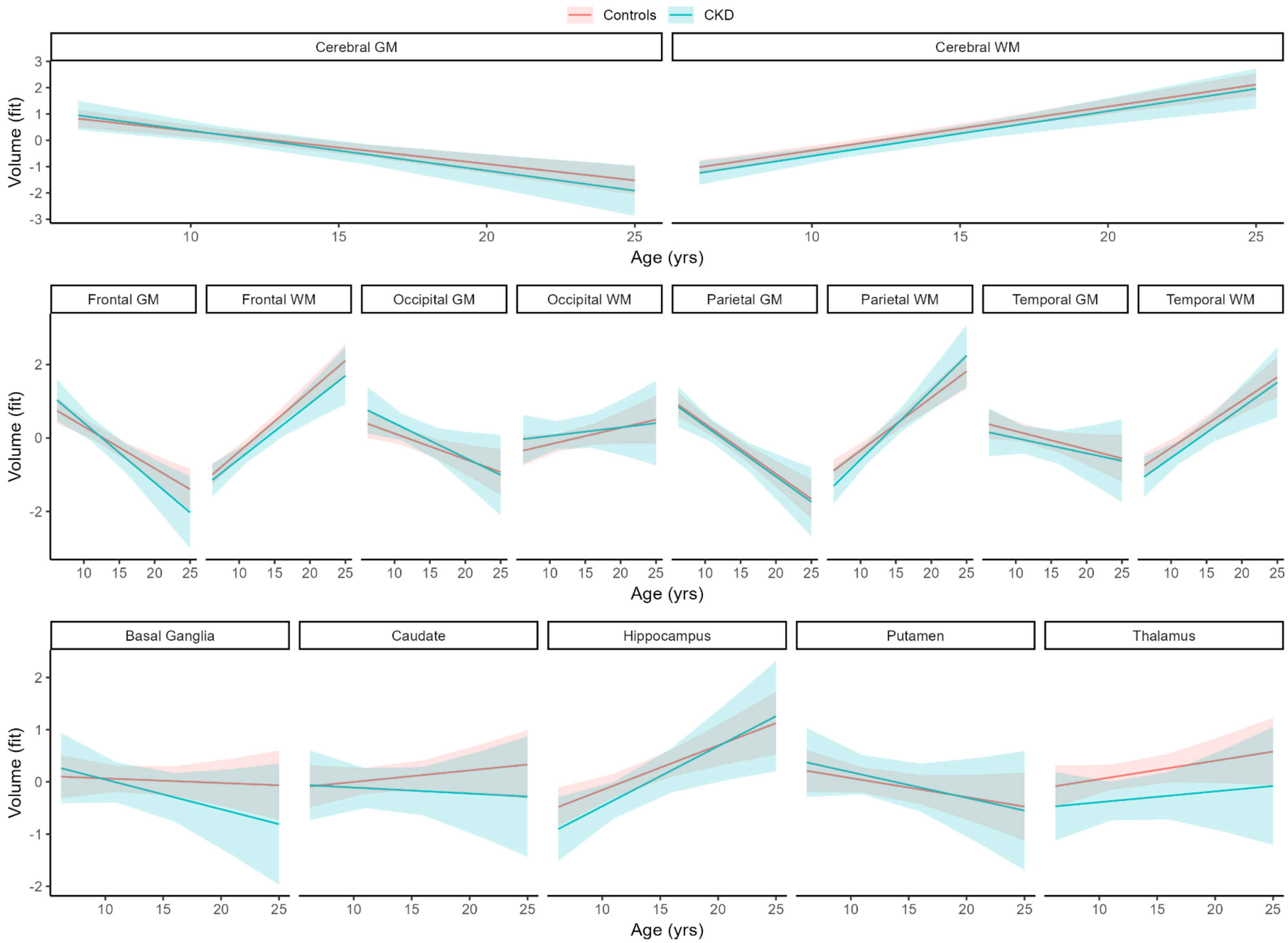

© 2024 van der Plas E et al. *JAMA Network Open*.

eFigure 3. Age-related changes (x-axes) across cerebellum lobes in control (pink) and patients with CKD (blue).

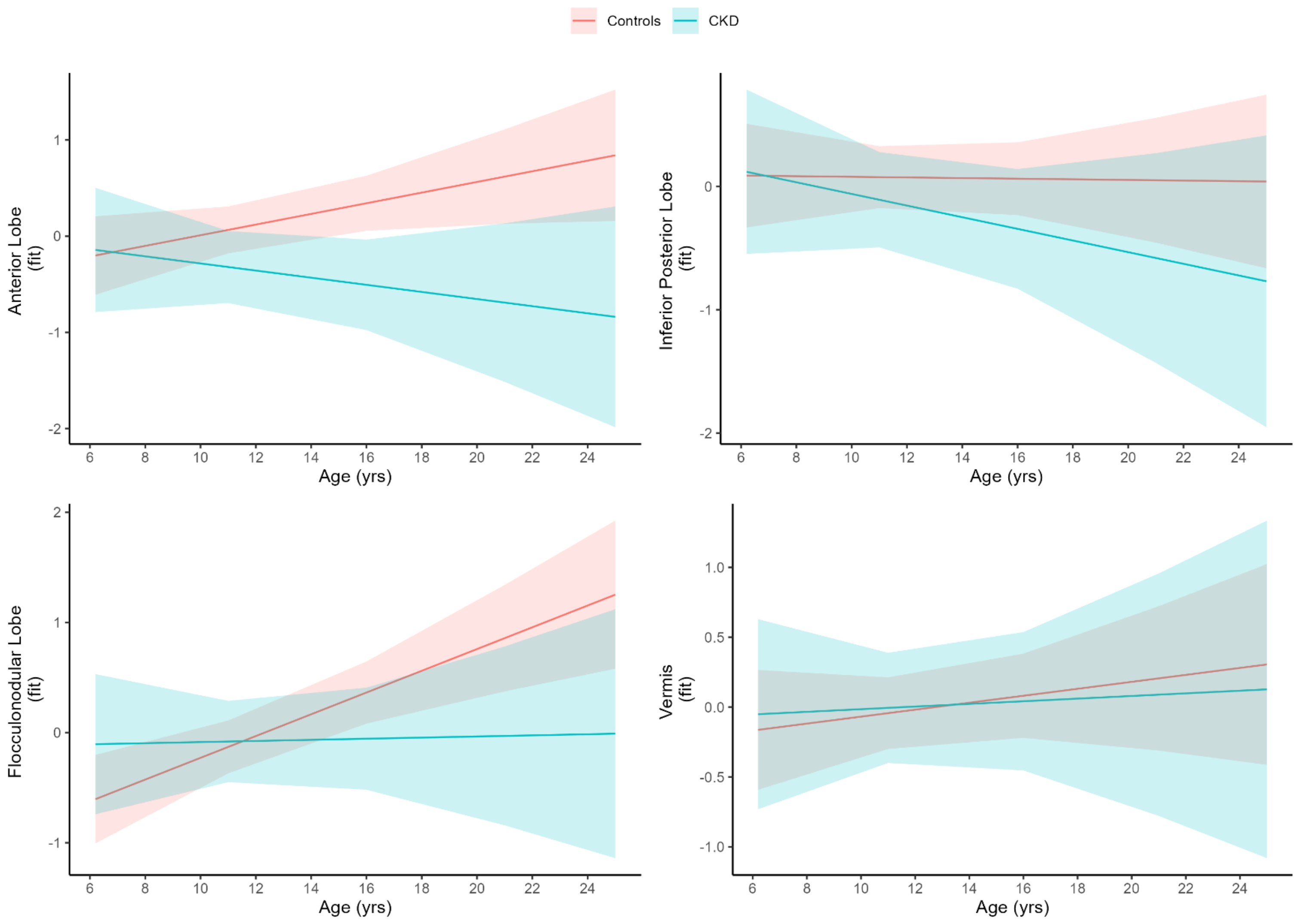

© 2024 van der Plas E et al. *JAMA Network Open*.

eFigure 4. Distribution of premature (pink) and term (blue) born participants across groups (x-axes) for regional volumes for which significant group differences were observed (y-axes). Panel A shows cerebellum gray matter volume, panel B depicts cerebellum white matter volume, and amygdala volume is shown in panel C.

Regional volumes are adjusted for ICV using the power proportion.

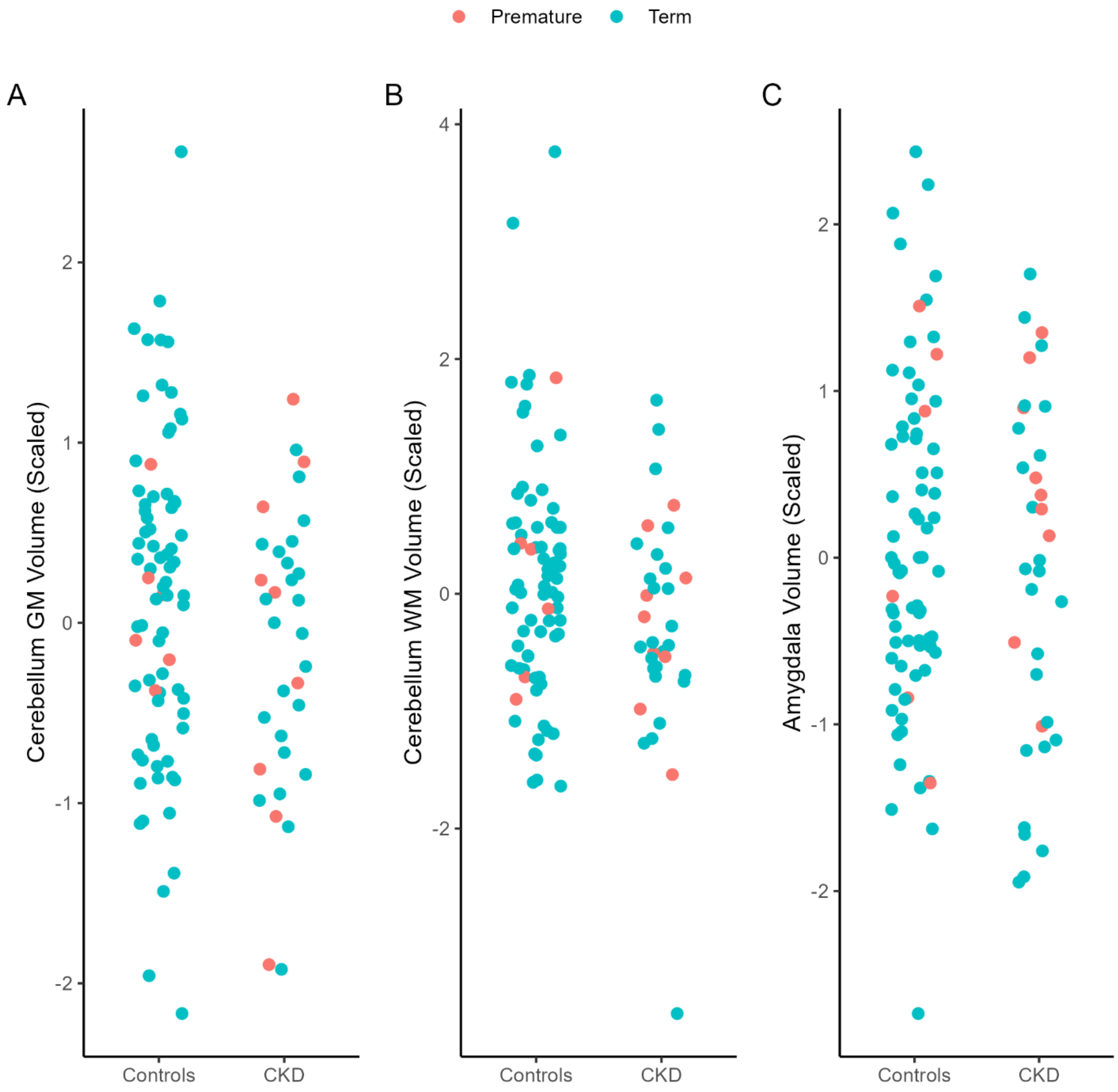

© 2024 van der Plas E et al. *JAMA Network Open*.

eFigure 5. Distribution of patients on blood pressure medication (blue) and those not on blood pressure medication (pink) for cerebellum gray matter volume (y-axis).

Distributions for controls and CKD are shown separately (x-axis). Each circle represents an observation, with blue representing individuals who took blood pressure medication. Unadjusted means and 95% confidence intervals of the means are included as well, showing that there is minimal difference in cerebellum gray matter volume in patients based on blood pressure medication.

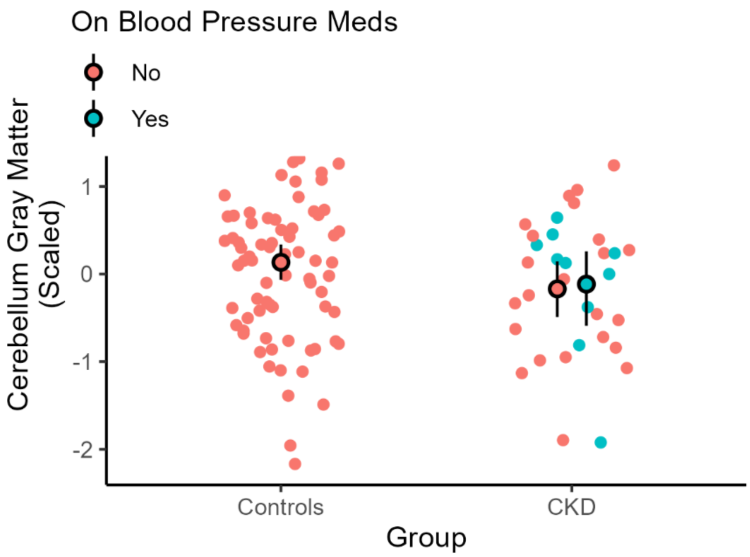

© 2024 van der Plas E et al. *JAMA Network Open*.

eFigure 6. Distribution of participants on anxiety or depression medication in the sample

Distributions of controls and CKD patients are shown separately (x-axis) for cerebellum gray matter volume (y-axis). Each circle represents an observation, with blue representing individuals who took anxiety/depression medication at the time of assessment. Unadjusted means and 95% confidence intervals of the means are included as well, showing that there is minimal difference in cerebellum gray matter volume in patients based on anxiety/depression medication intake.

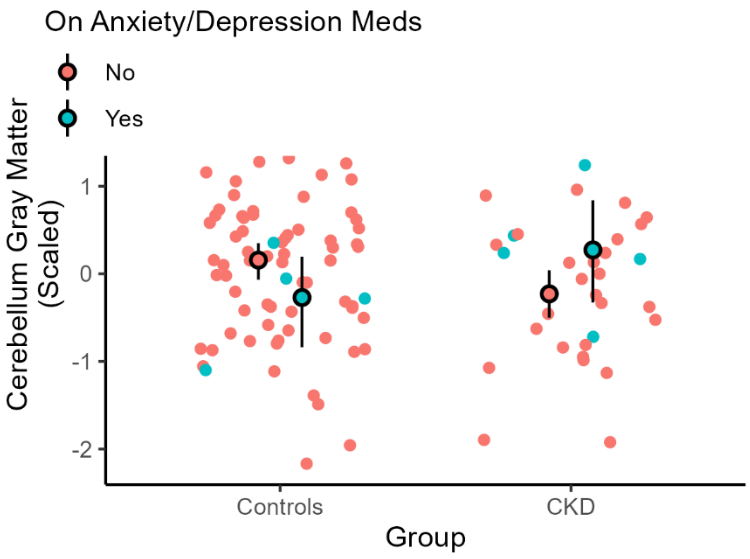

© 2024 van der Plas E et al. *JAMA Network Open*.
