# Supplementary material for: Age-Related Changes in Brain Structure in Pediatric Chronic Kidney Disease
Source: JAMA Netw Open. 2025 Feb 3;8(2):e2457601. doi: 10.1001/jamanetworkopen.2024.57601 (PMC11791706; doi:10.1001/jamanetworkopen.2024.57601)
Supplement: Supplement 2. — Data Sharing Statement [file jamanetwopen-e2457601-s002.pdf]

Data Sharing Statement

van der Plas. Age-Related Changes in Brain Structure In Pediatric Chronic Kidney Disease. *JAMA Netw Open*. Published February 03, 2025. doi:10.1001/jamanetworkopen.2024.57601

Data
Data available: Yes
Data types: Deidentified participant data, Data dictionary
How to access data: Deidentified participant data and data dictionary will be made available. Data requests must be sent to the corresponding author.
When available: With publication

Supporting Documents
Document types: None

Additional Information
Who can access the data: Researchers whose proposed use of data has been approved
Types of analyses: Data will be made available for a specific secondary research purpose
Mechanisms of data availability: Data will be made available after approval of a proposal for use and with a signed data use agreement
